# Basal cell adhesion molecule (BCAM) promotes mesothelial-to-mesenchymal transition and tumor angiogenesis through paracrine signaling

**DOI:** 10.1186/s12964-025-02128-9

**Published:** 2025-03-13

**Authors:** Suresh Sivakumar, Sonja Lieber, Raimund Dietze, Vanessa M. Beutgen, Eileen C. Sutor, Sophie Heidemann, Florian Finkernagel, Julia Teply-Szymanski, Andrea Nist, Thorsten Stiewe, Katrin Roth, Silke Reinartz, Johannes Graumann, Sabine Müller-Brüsselbach, Rolf Müller

**Affiliations:** 1 Translational Oncology, Center for Tumor Biology and Immunology (ZTI), Philipps University, Hans-Meerwein-Strasse 3, 35043 Marburg, Germany; 2 Institute of Systems Immunology, Center for Tumor Biology and Immunology (ZTI), Philipps University, Marburg, Germany; 3 Institute of Translational Proteomics, Biochemical/Pharmacological Centre, Philipps University, Marburg, Germany; 4 Core Facility Translational Proteomics, Philipps University, Marburg, Germany; 5 Genomics Core Facility, Philipps University, Marburg, Germany; 6 Institute of Molecular Oncology, Member of the German Center for Lung Research (DZL), Philipps University, Marburg, Germany; 7 Institute of Lung Health, Justus-Liebig University, Giessen, Germany; 8 Cell Imaging Core Facility, Center for Tumor Biology and Immunology (ZTI), Philipps University, Marburg, Germany

**Keywords:** Basal cell adhesion molecule (BCAM), Mesothelial-to-mesenchymal transition (MMT), Cancer cell migration, Ovarian cancer metastasis, Tumor angiogenesis

## Abstract

**Background:**

High expression of basal cell adhesion molecule (BCAM) is a hallmark of ovarian cancer (OC) progression. BCAM facilitates transcoelomic dissemination by promoting mesothelial cell clearance at peritoneal attachment sites of tumor cell spheroids. We investigated how BCAM mediates this effect and potentially drives other pro-metastatic functions.

**Methods:**

The impact of BCAM on the tumor cell secretome and the mesothelial cell phenotype was analyzed by affinity proteomics, bulk and single-cell RNA sequencing, life-cell and multiphoton microscopy, biochemical and functional in vitro assays as well as a murine tumor model. BCAM manipulation involved ectopic overexpression, inducible expression and treatment with soluble BCAM.

**Results:**

All forms of BCAM enhanced the secretion of cytokines that impact cell motility, mesenchymal differentiation and angiogenesis, including AREG, CXCL family members, FGF2, TGFB2, and VEGF. Notably, their levels in OC ascites were correlated with BCAM expression, and recombinant BCAM-induced cytokines triggered mesothelial-mesenchymal transition (MMT). Mesothelial cells undergoing MMT exhibited enhanced motility away from attaching tumor spheroids, leading to mesothelial clearance at spheroid attachment sites. BCAM-mediated MMT-associated transcriptional changes were also observed in subpopulations of omental mesothelial cells from OC patients, and were associated with poor survival. Consistent with the secretome data, BCAM induced endothelial tube formation in vitro and markedly promoted tumor angiogenesis in a mouse model.

**Conclusion:**

We have identified previously unknown functions of the BCAM-induced secretome potentially impacting distinct stages of OC metastasis. While BCAM’s impact on MMT may facilitate initiation of micrometastases, neo-angiogenesis is essential for tumor growth. Taken together with the observed clinical adverse association, our findings underscore the potential of BCAM as a therapeutic target.

**Supplementary Information:**

The online version contains supplementary material available at 10.1186/s12964-025-02128-9.

## Introduction

The basal cell adhesion molecule (BCAM), a member of the immunoglobulin superfamily [1–3]. is found in high concentrations in the tumor microenvironment (TME) of ovarian carcinoma (OC) and is linked to poor relapse-free survival (RFS) [4]. Two structurally distinct membrane-bound forms of BCAM have been identified: the full-length version BCAM1 and the alternatively spliced BCAM2 isoform lacking a majority of the cytoplasmic domain [5]. Moreover, soluble sBCAM produced by matrix metalloproteinases has been identified [6, 7]. sBCAM is abundant in OC ascites, a critical compartment of the TME that plays a key role in peritoneal metastatic spread of OC cells [4, 7].

BCAM binds to the laminin α5 (LAMA5) chain, a component of laminin trimers in the extracellular matrix (ECM), such as LN-511 [1, 8–12], thereby interfering with the interaction of laminin and β1 integrins [13]. This interaction influences tumor cell adhesion and migration with distinct effects across individual cell types [7, 13–17]. In our recent work [7], we identified new functions of BCAM in OC: (i) an inhibitory effect on the compaction of tumor cell spheroids, promoting their dispersion within a 3D matrix; and (ii) induction of mesothelial clearance at spheroid attachment sites, a crucial step in the metastatic trans-mesothelial invasion of tumor cells [18].

Mechanistically, all forms of BCAM interfere with the interaction between LAMA5 and integrin β1, resulting in reduced adhesion of single cells to an LN-511 matrix [7]. This reduction in adhesion potentially represents an anti-metastatic mechanism. However, OC cell adhesion to collagen, rather than laminin, is a primary driver of peritoneal dissemination [19–21]. Notably, BCAM does not affect adhesion to COL1, suggesting that its inhibitory effect on single-cell adhesion to LN-511 may have limited significance in the context of OC [7]. Importantly, the LAMA5/integrin-β1 interaction is also essential for spheroid compaction, and BCAM’s disruption of this interaction compromises spheroid integrity. This disruption likely contributes to peritoneal metastasis by enhancing the dispersion of cancer cell spheroids at target sites. This conclusion is supported by an observed increase in peritoneal colonization by BCAM-transduced OC cells in both explanted omentum and a mouse model [7].

The observation that all BCAM isoforms exert comparable effects on OC spheroid compaction suggests BCAM to function as a ligand or decoy rather than as a receptor. This idea aligns with the reported inhibitory effect of either soluble LN-511 or an activating integrin β1 antibody on BCAM’s impact on spheroid compaction [7]. These findings further imply that other reported interactions of BCAM, such as with α3β1, α4β1, α6β1 or α7β1 [13, 22, 23] are unlikely to be involved. Consequently, the specific mechanisms and signaling pathways underlying the function of BCAM in spheroid formation remain unclear and may involve interactions with unidentified molecules beyond laminins and integrins.

Several mechanisms have been proposed to compromise the integrity of the mesothelial cell layer and facilitate OC cell invasion [24]. These include (i) the clearance of mesothelial cells through myosin-dependent mechanical forces exerted by tumor cells [25], (ii) changes in mesothelial cell morphology due to mesenchymal transformation triggered by mediators in the TME [26], (iii) the induction of mesothelial cell senescence [27], and (iv) the induction of apoptosis via death ligands [28, 29], TGFβ signaling [30] or extracellular vesicles [31–33]. Our previous work has shown that BCAM facilitates both metastasis formation and mesothelial clearance at spheroid attachment sites [18]. However, it remains unclear whether these effects are linked to the aforementioned mechanisms or arise from an as-yet unidentified mode of action.

In this study, we have therefore specifically explored the mechanisms underlying BCAM-triggered mesothelial clearance as well as other potentially pro-metastatic functions. To achieve this, we employed a multi-omics approach alongside microscopic analyses and functional assays as well as a mouse tumor model. These studies identified BCAM-induced MMT as the primary mechanism driving mesothelial clearance in a paracrine manner and uncovered the induction of tumor angiogenesis as a further previously unknown biological function of BCAM. Contrary to the reported pro-tumorigenic functions of BCAM, the effects described here were independent of LAMA5 and integrin β1 interactions, consistent with a novel mode of action.

## Materials and methods

### Patient samples

Ascites and omentum tissue with metastatic lesions were collected from patients with ovarian high-grade serous carcinoma undergoing primary surgery at the University Hospital in Marburg. The acquisition and analysis of human specimens was approved by the local ethics committee (reference number 205/10). Donors provided their written consent in accordance with the Declaration of Helsinki.

### Cell cultures

OVCAR4 and OVCAR8 cells were obtained from the NIGMS Human Genetic Cell Repository of the NIH (Bethesda, Maryland, USA). OVCAR-8 cells overexpressing ectopic BCAM1 or BCAM2 (BCAM-OE cells: BCAM1-2, BCAM1-8, BCAM2-1 and BCAM2-3) corresponding vector control cells (clones pcDNA-3, pcDNA-6 and pcDNA-15) and OVCAR-8 cells with genetically inactivated BCAM alleles (BCAM-KO cells) have previously been described [7]. All OVCAR cell lines were cultured in RPMI 1640 (Cat. #61870044; Thermo Fisher Scientific, Darmstadt, Germany) supplemented with 10% FBS (Cat. #FBS-LE-12 A/RES1822; Capricon Scientific, Ebsdorfergrund, Germany).

Primary OC cell cultures were established from ascites-derived tumor cell spheroids (< 30 μm) obtained from a patient with histological grading G3 as described [34, 35]. These cells, subsequently referred to as OCMI-91s cells, were cultured in OCMI medium with 5% FCS and were used for maximum 20 passage [34].

Human peritoneal mesothelial cells (HPMCs) were isolated from tumor-free regions of the omentum of OC patients as previously described [36]. In brief, omentum was digested with trypsin for 30 min, followed by MACS depletion of CD45^+^ and EpCAM^+^ cells. Mesothelial cells were cultured in OCMI/5% FCS [34, 35] for 3–5 passages prior to use. Human umbilical vein endothelial cells (HUVECs) [37] were cultured using HUVEC Endothelial Growth Media (Cat. #C-22011; PromoCell, Heidelberg, Germany).

### Establishment and culturing of doxycyclin-inducible cells

OVCAR-8 cells with disrupted BCAM alleles (BCAM_KO cells) [7] were transfected with the pTetOne vector (Cat. #634301; Takara Bio Europe, Saint-Germain-en-Laye, France) containing either BCAM1 (Transcript 1, Ref. Seq - NM_005581.4) or BCAM2 (Transcript 2, Ref. Seq - NP_001013275.1). The cells were co-transfected with a linear selection marker containing puromycin resistance, using the Xfect transfection reagent according to the manufacturer’s instructions. Cells were selected in the presence of puromycin (Sigma-Aldrich; Taufkirchen, Germany; Cat. #P8833) at a concentration of 0.25 µg/ml. Stable clones were then analyzed for BCAM expression by immunoblotting and FACS analysis. The clones were cultured in RPMI 1640 (Life Technologies, Darmstadt, Germany) supplemented with 10% Tet System Approved FBS (Takara Bio Europe; Saint-Germain-en-Laye, France; Cat. #631106) and 1% Penicillin-Streptomycin (Cat. #P0781; Sigma-Aldrich, Taufkirchen, Germany). For BCAM induction, doxycycline was added to the culture medium at a concentration of 1 µg/ml (Cat. #D9891-1G; Sigma-Aldrich, Taufkirchen, Germany) for up to 12 days as indicated in the corresponding figure legends. For kinetic experiments lasting longer than 72 h, the medium was replaced every 96 h, with doxycycline replenished at each change.

### Antibodies

Monoclonal anti-human BCAM antibody (Cat. #MAB1481) and polyclonal anti-human BCAM antibody (Cat. #AF148) were obtained from R&D Systems/Bio-Techne. Anti-ZO1-AlexaFluor 488 antibody (Cat. #33-9100) was purchased from Invitrogen, anti-CDH1 antibody (Cat. #ab219332) from Abcam, anti-FN1 antibody (Cat. #26836) from Cell Signaling Technology, and anti-β-actin (clone AC-15; Cat. #A5441) from Sigma-Aldrich. Anti-CD45-PE (clone 30-F11; Cat. #553081), anti-CD45-APC (clone 30-F11; Cat. #559864), anti-CD31-PE (Cat. #553373), and anti-VCAM1/CD106 (clone 429, Cat. #561615) antibodies were procured from BD BioSciences. Anti-CD45-AlexaFluor 647 (clone 30-F11, Cat. #103124) was obtained from BioLegend, and anti-CD31-Vio 667 (clone REA784, Cat. #130-128-736) from Miltenyi Biotec.

### Other materials

Recombinant Fc-BCAM produced in a mouse myeloma cell line (Cat. #148-BC) and negative control Fc from IgG1 (Cat. #110-HG) were obtained from R&D Systems/Bio-Techne (Wiesbaden, Germany). Recombinant Human Laminin 511 (Cat. #LN-511) was purchased from BioLamina (Sundbyberg, Sweden) and rat tail collagen I (Cat. #A1048301) from Thermo Fisher Scientific. Cell Tracker Green CMFDA (Cat. #C2925), Cell Tracker Orange CMTMR (Cat. #C2927), Cell Tracker Blue CMAC (Cat. #C2110), and Cell Tracker Deep Red (Cat. #C34565), were from Thermo Fisher Scientific, and Biotracker 555 Orange (Cat. #SCT107) from Merck (Darmstadt, Germany). Recombinant protein human amphiregulin produced in HEK293 cells (Cat. #HY-P7002) was obtained from Biozol (Eching, Germany, HumanKine^®^ recombinant human FGFbasic-TS protein produced in HEK293 cells (Cat. #HZ-1285) from Proteintech (Manchester, UK), recombinant human endothelin 1 protein produced in HEK293 cells (Cat. #TP302217) was from Origene EU (Herford, Germany), and recombinant human TGFβ1 produced in HEK293 cells (Cat. #T7039-2UG) was from Th. Geyer (Renningen, Germany). 2-(N-(7-Nitrobenz-2-oxa-1,3-diazol-4-yl)amino)-2-desoxyglucose (2-NBDG) was purchased from R&D Systems/Bio-Techne.

### siRNA-mediated interference

siRNA transfection was performed in OVCAR4 cells cultured in RPMI plus 10% FCS using the Lipofectamine 2000 (Invitrogen/Thermo Fisher Scientific; Cat. #11668027) reagent according to the manufacturer’s protocol. BCAM siRNA-mediated interference was performed using three different siRNA oligonucleotides (Sigma–Aldrich; Taufkirchen, Germany): BCAM #1 (5′‐GAGACUACGUGUGCGUGGU‐3′), BCAM #2 (5′‐GGAU UACGACGCGGCAGAU‐3′), BCAM #3 (5′‐ CAGAGCUAAAGACAGCGGA ‐3′). MISSION siRNA Universal Negative Control #1 from Sigma–Aldrich (Taufkirchen, Germany) was used as a control. Cells were harvested 72 h after transfection.

## Quantification of BCAM by flow cytometry

Cells were detached from cell culture dishes using Accutase cell dissociation solution (Cat. #A6964; Sigma Aldrich), washed and incubated with monoclonal anti-human BCAM or isotype control antibodies (R&D Systems/Bio-Techne) followed by FITC-labeled anti-mouse IgG (Cat. #11-4011-85; eBioscience/Thermo Fisher Scientific). Cells were analyzed by flow cytometry using a FACS Canto II instrument using Diva Software (BD Biosciences, Heidelberg, Germany). Results were calculated as percentage of positive cells and mean fluorescence intensities (MFI). Cell death was assessed by propidium iodide staining.

### Immunoblotting

Immunoblots were performed according to standard protocols using the primary antibodies listed above in combination with secondary α-rabbit IgG HRP‐linked polyclonal antibody (Cat. #7074, RRID: AB_2099233; Cell Signaling Technology); α‐mouse IgG HRP‐linked polyclonal antibody (Cat. #7076, RRID: AB_330924; Cell Signaling Technology) and α-goat IgG HRP-linked polyclonal antibody (Jackson ImmunoResearch Labs/Dianova, Hamburg, Germany). Imaging and quantification were carried out using the ChemiDoc MP system and Image Lab software version 5 (Bio-Rad; Feldkirchen, Germany). Total protein staining was performed directly after the transfer with PierceTM Reversible Protein Stain Kit (Cat. #24585; Thermo Fisher) according to the kit protocol.

### Preparation of conditioned medium (CM)

Cells were seeded in 10 cm dishes at a density of 3.5 × 10⁶ cells per dish, and the medium was replaced with serum-free medium after 24 h. BCAM-OE cells were incubated for an additional 48 h. CM was collected, centrifuged at 300xg for 5 min to remove dead cells, and further clarified by centrifugation at 1500xg for 5 min. TET-BCAM-1-3 cells ± Dox were cultured for 96 h in serum-free medium prior to collection of CM. CM from OVCAR-4 cells was harvested 72 h after siRNA transfection.

### ELISA

The levels of AREG, FGF2, VEGF-A, VEGF-B, CXCL1, and EDN1 in CM were quantified by enzyme-linked immunosorbent assay (ELISA), using the following kits: AREG (Cat. #DAR00, R&D Systems/Bio-Techne), FGF2 (Cat. #DFB50, R&D Systems/Bio-Techne), VEGF-A (Cat. #BMS277-2, Life/Thermo Fisher), VEGF-B (Cat. #CSB-E04758H-96, Biozol; Eching, Germany), CXCL1 (Cat. #EHCXCL1, Life/Thermo Fisher), and EDN1 (Cat. #DET100, R&D Systems/Bio-Techne). ELISAs were performed according to the manufacturer’s instructions.

### Angiogenesis assay

The µ-Slide 15 Well 3D plate from ibidi (Cat. #81506; Ibidi, Gräfelfing, Germany) was precoated with 10 µL of Matrigel Growth Factor Reduced Basement Membrane Extract (Cat. #356230; Corning Life Sciences, Kaiserslautern, Germany) on ice and incubated at 37 °C for a minimum of 30 min under a 5% CO_2_ humidified atmosphere for polymerization. HUVECs were stained with BioTracker 555 Orange Cytoplasmic Membrane dye prior to resuspension in Endothelial Cell Basal Medium (EBM) as negative control or EBM with CM at a 1:3 ratio. Cells in Endothelial Cell Growth Medium 2 served as positive control. These cells were seeded at a density of 8000 cells/well in a final volume of 50 µL and incubated at 37 °C under 5% CO_2_. Images were acquired at 2 h-intervals using a Leica SP8i confocal microscope (Leica, Wetzlar, Germany) with 5x magnification. Image analysis was performed by using the Angiogenesis Analyzer plugin for ImageJ/Fiji software. For the quantification of tube formation, total branching length was used, and the final tube length was plotted in µm.

### Affinity-based proteomics

Supernatant from BCAM-OE cells was analyzed using the Olink Explore 3072 platform at the Core Facility Translational Proteomics at the Medical Department of Philipps-University Marburg (UMR), following the Olink protocol (v4.0, 2024-04-16) with adjustments for cell culture supernatant analysis. All jointly analyzed samples were randomized and plated on 96-well plates. Samples were processed in one batch. Next-Generation Sequencing (NGS) of the generated libraries was performed at the Genomics Core Facility of the Department of Medicine at UMR. Olink Explore uses Proximity Extension Assays (PEA) technology [38] and has been optimized for high-throughput analysis by NGS [39]. In brief, the PEA immunoassay uses two matched antibodies per target, binding simultaneously to different epitopes on the protein. The antibodies are covalently labeled with complementary oligonucleotide probes that only hybridize when the correct pair of matching antibodies is in close proximity. The resulting short dsDNA sequences contain an assay specific barcode and are pre-amplified by an initial PCR. In a second PCR, additional DNA tags providing information on the respective sample are added to the barcodes. This coding allows for the parallel measurement of ~ 3,000 proteins.

Protein levels are expressed as Normalized Protein eXpression (NPX; Olink-provided arbitrary unit in log2 scale). For mediators present in more than one panel (CXCL8, IDO1, IL6, LMOD1, SCRIB, TNF) mean NPX values were used in subsequent bioinformatic analyses. Fold change (FC) values (2^∆NPX^) were calculated as the median of *n* = 5 biological replicates. False discovery rates (FDR) were determined by applying the Benjamini-Hochberg method to nominal p values determined by unpaired t test of FC values.

### Bulk RNA-Seq

Bulk RNA was transcribed into an Illumina-compatible library using the Lexogen Quantseq FWD mRNA-seq v2 Library Prep Kit with UDI (Lexogen, Vienna) according to manufacture’s instructions and sequenced on an Ilumina NextSeq 550 at the local Genomics Core facility. Single end RNA-Seq reads were aligned using STAR^43^ (version 2.61d) against Ensembl 96 [40]. Reads were quantified within the exons of protein coding transcripts and normalized to ‘counts per million’. Genes with expression values below 1 CPM were excluded from further analyses. Differential expression was estimated by unpaired t test and filtering for FDR < 0.05, FC > 2 (BCAM-OE) or FC > 1.5 (TET-BCAM) determined by applying the Benjamini-Hochberg method. RNA-seq data was anonymized using BAMBoozle and deposited at EBI Array Express under accession numbers E-MTAB-14,411 and E-MTAB-14,412.

### Single-cell RNA sequencing of patient-derived mesothelial cells

Single-cell RNA sequencing (scRNA-Seq) data of omental mesothelial cells from HGSC patients generated in a previous study by our laboratory [41] (EBI Array Express; E-MTAB-13498) were reanalyzed for the expression of a BCAM-induced MMT-associated genes. Briefly, samples were analyzed using the Rhapsody single-cell capture system (Becton, Dickinson and Company) by targeted sequencing on an Illumina NextSeq 550 by the local Genomics Core Facility, and aligned and quantified using STARSolo as described [42]. The reference consisted of the Homo Sapiens genome sequence GRCH38.p13 retrieved together with the used gene models from Ensembl 108, extended with the Rhapsody sample tags for Homo sapiens. Cell barcodes were retrieved from published data [43, 44]. The targeted gene set consisted of the Becton Dickinson Rhapsody Onco-BC Targeted Panel (https://scomix.bd.com/hc/article_attachments/13766899704717) and a panel of genes regulated by cytokines in mesothelial cells [41]. Of these, *n* = 38 were identified as upregulated by the BCAM secretome through bulk RNA-Seq (Table S1; FC > 2; FDR < 0,05: median CPM > 1). Genes expressed in ≥ 100 cells (labeled red in Table S1; *n* = 24) were included in subsequent analyses.

Following UMAP-based embedding [45–47] Louvain clustering [48] clustering and cell type annotation using SCSA [49], the mesothelial cell cluster was identified by expression of mesothelial markers genes *(ITLN1, HP*, *UPK3B)* [41]. Due to the potential occurrence of MMT, cells expressing fibroblast marker genes *(DCN, FBLN1 MMP2)* were also included. Further filtering was performed to exclude cells expressing epithelial or immune-cell-selective markers as described [41]. The cluster was subsequently analyzed for the expression of BCAM-induced genes (identified through bulk RNA-Seq) by quantifying the number of expressed genes per cell. The expression of mesothelial and/or mesenchymal marker genes was assessed using the same approach.

### Mouse model

The peritoneal OC cell dissemination mouse model has previously been described in detail [7]. Briefly, 500,000 cells were seeded per well in 24-well ultra-low attachment plates (Merck; Cat #CLS3473-24EA) and cultured in RPMI medium supplemented with 10% fetal calf serum (FCS) to induce spheroid formation. After 72 h, spheroids from two wells were combined and injected intraperitoneally into BALB/c-nude mice. After 28 days, the omental tumor masses and their vascularization were examined. To facilitate the microscopic visualization of tumor cells, 100 µl of a 5 mM solution of the glucose analog 2-NBDG was administered via tail vein injection. Mice were euthanized 30 min after the 2-NBDG injection, and the omentum was dissected. Whole mounts were then stained for CD45 and CD31 using immunofluorescence and analyzed by multiphoton microscopy according to the published protocol [7].

To quantify the tumor vasculature multiphoton images were processed by a machine learning-based algorithm (Imaris 10.0 with filament module; Bitplane-Oxford Instruments, Wiesbaden, Germany). The analysis was restricted to subregions with tumor cells without milky spots using the “autopath (loops) no soma no spines” setting along with definition of smallest and maximum diameter of vessels and intensity thresholds. To account for variations in tumor size, regions were normalized to a volume of 5 × 10^7^ µm^3^.

### Statistical analyses of and functional annotations

Comparative data were statistically analyzed by paired or unpaired Student’s *t*-test (two-sided, unequal variance), or by two-way ANOVA, as indicated in the Figure legends. Quantification of tumor blood vessels was analyzed by one-way ANOVA with Bonferroni posttest. Statistical significances are indicated as follows: *p < 0.05; **p < 0.01; ***p < 0.001; ****p < 0.0001. Box plots were generated using the Seaborn boxplot function with Python and depict medians, upper and lower quartiles, range and outliers. Fitted lines were plotted using the Python numpy.polyfit function. Gene ontology enrichment analysis was carried out using the Gene Ontology (GO) Biological Process Complete resource [50] at http://geneontology.org.

## Results

### The BCAM-induced OC cell secretome

**Fig. 1:**
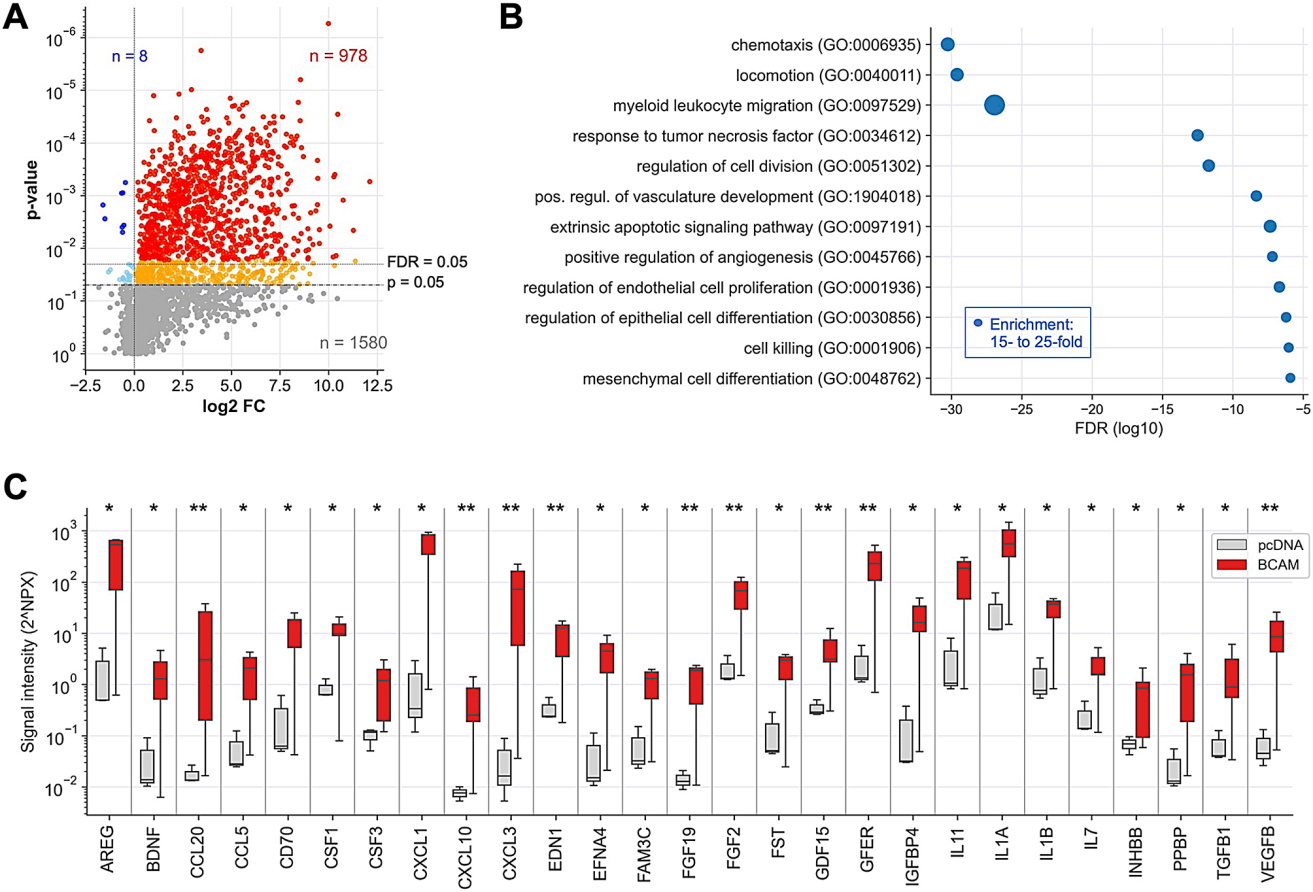
The BCAM-induced secretome of OVCAR-8 cells. CM from BCAM-OE cells and vector control cells was collected 48 h after medium change and analyzed by PEA-based affinity proteomics. **(A)** Volcano plots depicting the BCAM-induced fold change (FC) in signal intensities (log2). Blue: downregulated proteins (light blue: nominal *p* < 0.05; dark blue FDR < 0.05). Orange/red: upregulated proteins (orange: *p* < 0.05; red: FDR < 0.05). Grey: proteins not significantly affected (*n* = 1,580). Dashed horizontal lines indicate the significance thresholds. The numbers of downregulated (blue) and upregulated (red) proteins (FDR < 0.05) are shown at the top. **(B)** GO term enrichment analysis of BCAM-induced cytokines (*n* = 69; FC > 3; FDR < 0.1). The analysis was performed using the “Biological Process Complete” function of Gene Ontology Resource [50]. The plot shows the 12 non-redundant terms with the lowest FDR. The size of the filled circles indicated the fold enrichment. **(C)** Boxplot depicting signal intensities (2^NPX^) for BCAM-regulated cytokines (FC > 10; FDR < 0.05) associated with EMT or angiogenesis according to the GeneCards database [53, 54]. The analyses were performed with *n* = 11 samples of BCAM-OE cells (clones BCAM1-2, BCAM1-8, BCAM2-1, BCAM2-3) and *n* = 3 samples of vector control cells (clone pcDNA-3). The plot shows the median (line), upper and lower quartiles (box), range (whiskers). **FDR < 0.01; ns, not significant by unpaired t test and Benjamini-Hochberg adjustment

To elucidate the BCAM-induced secretome in OC cells, we conducted PEA-based affinity proteomics (Table S2) on conditioned medium (CM) from BCAM-overexpressing OVCAR-8 cells (BCAM-OE: clones BCAM1-2, BCAM1-8, BCAM2-1, BCAM2-3) and CM from control cells transduced with an empty expression vector (clone pcDNA-3). As illustrated by the volcano plot in Fig. 1A, this analysis identified *n* = 978 proteins that were significantly (FDR < 0.05) upregulated in BCAM-OE cells, while only *n* = 8 proteins were downregulated.

Among the most significantly BCAM-induced proteins predicted as “secreted” in the Human Protein Atlas [51] (FDR < 0.05; *n* = 229), *n* = 61 were cytokines and growth factors. Functional annotation of these proteins, using the “Biological Process Complete” function of the Gene Ontology Resource [50] highlighted significant enrichment in biological processes related to cell motility/migration, proliferation, apoptosis, vasculature development/angiogenesis, and epithelial/mesenchymal differentiation (Table S3). Figure 1B presents a summary of the most significant, non-redundant terms describing specific biological processes. The association with motility/migration and epithelial/mesenchymal differentiation suggests a link between BCAM and epithelial-to-mesenchymal transition (EMT) [52], which is further supported by the observation that *n* = 145 of the BCAM-induced secreted proteins have previously been associated with EMT according to the GeneCards database [53, 54].

The most highly induced proteins (*n* = 27; FC > 10; FDR < 0.05) linked to EMT and/or angiogenesis are depicted in the boxplot in Fig. 1C, including amphiregulin (AREG), several CCXL chemokines, endothelin 1 (EDN1), fibroblast growth factor 2 (FGF2), transforming growth factor β (TGFB) and vascular endothelial growth factor B (VEGFB). The functional annotation in panel B also suggests a connection between BCAM and angiogenesis-promoting proteins, as shown for members of the FGF, IL1, TGFB and VEGF families in Fig. 1C, which intersect with inducers of EMT.

All previously described functions of BCAM in tumor cells [7, 13–17]– including effects on cell adhesion, migration, and spheroid compaction– are dependent on BCAM’s role as a decoy receptor for LAMA5, interfering with the interaction of LN-511 laminin trimers with integrin β1 [1, 8–13]. For instance, the inhibitory effect of BCAM on OC cell migration in vitro required the presence of an LN-511 matrix, and its influence on spheroid compaction was inhibited by an activating antibody targeting integrin β1. In contrast, as illustrated in Fig. S1A-C, the secretion of AREG, CXCL1, and FGF2 by BCAM1-2 cells were independent of LN-511 coating and was unaffected by an activating integrin β1 antibody. Furthermore, collagen coating had no effect on AREG secretion (Fig. S1A), suggesting that BCAM-induced cytokine production is matrix-independent. This contrasts with BCAM’s impact on adhesion which is LN-511 dependent (Fig. S1D) [7], pointing to a novel mechanism mediating BCAM-induced cytokine secretion.

### Validation of affinity proteomics data

**Fig. 2:**
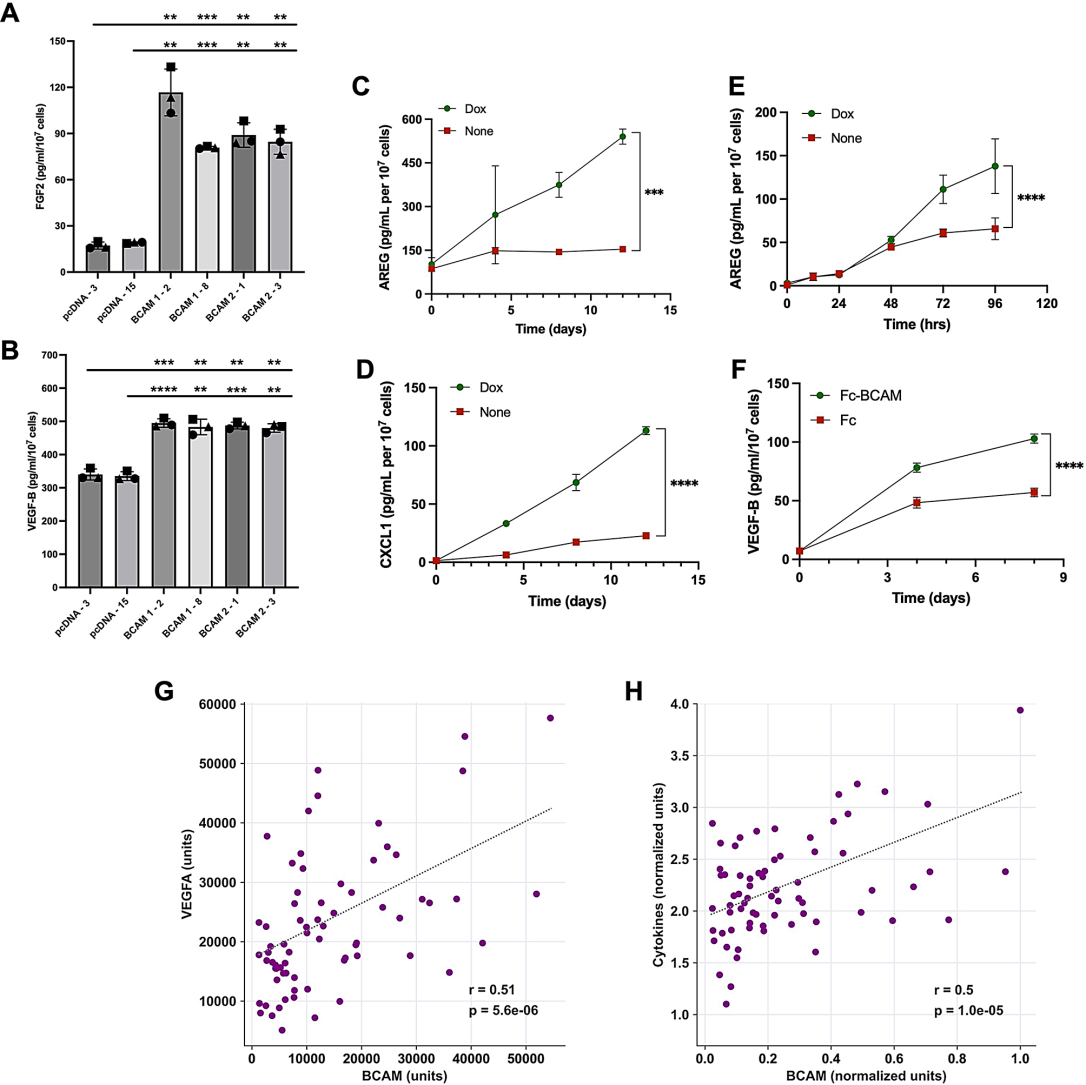
Validation of PEA-based affinity proteomics data. **(A, B)** Comparative analysis of CM from 4 different BCAM-OE clones (BCAM1-2, BCAM1-8, BCAM2-1, BCAM2-3) and two vector control clones (pcDNA-3, pcDNA-15). CM was collected 48 h after medium change (serum-free), and FGF2 (A) and VEGF-B levels were determined by ELISA (*n* = 3 biological replicates for each clone). **p < 0.01, ***p < 0.001, ****p < 0.0001 by unpaired t test. ns, not significant. **(C, D)** Kinetics of AREG and CXCL1 accumulation in CM from TET-BCAM-1-3 cells in the presence and absence of Dox for 12 days. **(E)** Kinetics of AREG accumulation in CM from TET-BCAM-1-3 cells in the presence and absence of Dox for 120 h. Medium and Dox were replaced every 96 h; CM was collected 96 h after medium change (serum-free) on day 8. **(F)** Kinetics of VEGF-B accumulation in CM from OVCAR-8 cells treated with Fc-BCAM (1 µg/ml) or control Fc (0.33 µg/ml; equimolar to Fc-BCAM) for 8 days. Significance in panels C-F was tested by 2-way ANOVA (***p < 0.001, ****p < 0.0001); *n* = 3 replicates for each timepoint. **(G)** Correlation of BCAM and VEGFA concentrations in OC ascites from b = 70 patients. The analysis is based on our previously published affinity proteomics (SomaScan) dataset [4]. **(H)** Correlation of normalized BCAM and cumulative protein-wise normalized levels of ANGPTL4, AREG, CXCL1, CXCL10, FGF2, VEGFA and VEGFC. r: Pearson correlation; p: significance of correlation, dashed lines: least square best-fit

We proceeded to verify the affinity proteomics results in various experimental systems:

- The findings for AREG, CXCL1, EDN1, FGF2, VEGFA and VEGFB were confirmed using E Fig. (Fig. 2 and S2).

- We developed a doxycycline-inducible (Dox) system in OVCAR-8 cells with genetically disrupted BCAM alleles (BCAM-KO cells), referred to as TET-BCAM-1-3 cells. These cells do not express detectable levels of BCAM1 in the absence of Dox, but show strong induction upon Dox treatment, as demonstrated by immunoblotting (Fig. S3A) and flow cytometry (Fig. S3B). Immunofluorescent staining confirmed that the induced protein localized correctly to the plasma membrane (Fig. S3C). Dox treatment resulted in a highly significant and progressively increasing induction of (Fig. 2C) and C (Fig. 2D) over 12 days. Additionally, short-term kinetics for AREG revealed induction by Dox within 72 h (Fig. 2E).
- Treatment of BCAM-KO cells with soluble Fc-BCAM led to a significant, time-dependent induction of VEGF-B compared to control Fc treat (Fig. 2F).
- A significant decrease in AREG, CXCL1, and FGF2 secretion in OVCAR-4 cells (which express high levels of BCAM) was observed following treatment with BCAM siRNA (Fig. S4).
- The induction of AREG and FGF2 by Fc-BCAM was replicated in patient-derived OC cells (OCMI-91s cells; Fig. S5).

We next sought to explore potential connections between BCAM expression and the levels of BCAM-induced cytokines in the OC TME. To this end, we utilized our previously published dataset of protein concentrations in the ascites of *n* = 70 OC patients, determined by SomaScan-based affinity proteomics [4]. As illustrated in Fig. 2G, the level of VEGFA, which is induced 177-fold in CM from BCAM-overexpressing (BCAM-OE) tumor cells (Table S2), showed a significant correlation with the concentration of soluble BCAM (Pearson *r* = 0.51; *p* = 5.6 × 10^− 6^). A similar correlation (Pearson *r* = 0.50; *p* = 10^− 5^; Fig. 2H) was observed for the cumulative normalized levels of several BCAM-induced cytokines and growth factors, which are relevant to our studies of epithelial-mesenchymal transition (EMT) and angiogenesis below. These include ANGPTL4 (induced 16-fold in BCAM-OE cell CM; Table S2), AREG (1,074-fold), CXCL1 (2,474-fold), CXCL10 (33-fold), FGF2 (51-fold), VEGFA (177-fold), and VEGFC (20-fold). These findings are compatible with a regulatory role of BCAM in modulating the tumor secretome in OC patients.

### BCAM-induced mesenchymal transformation disrupts the mesothelial monolayer and triggers mesothelial clearance

**Fig. 3:**
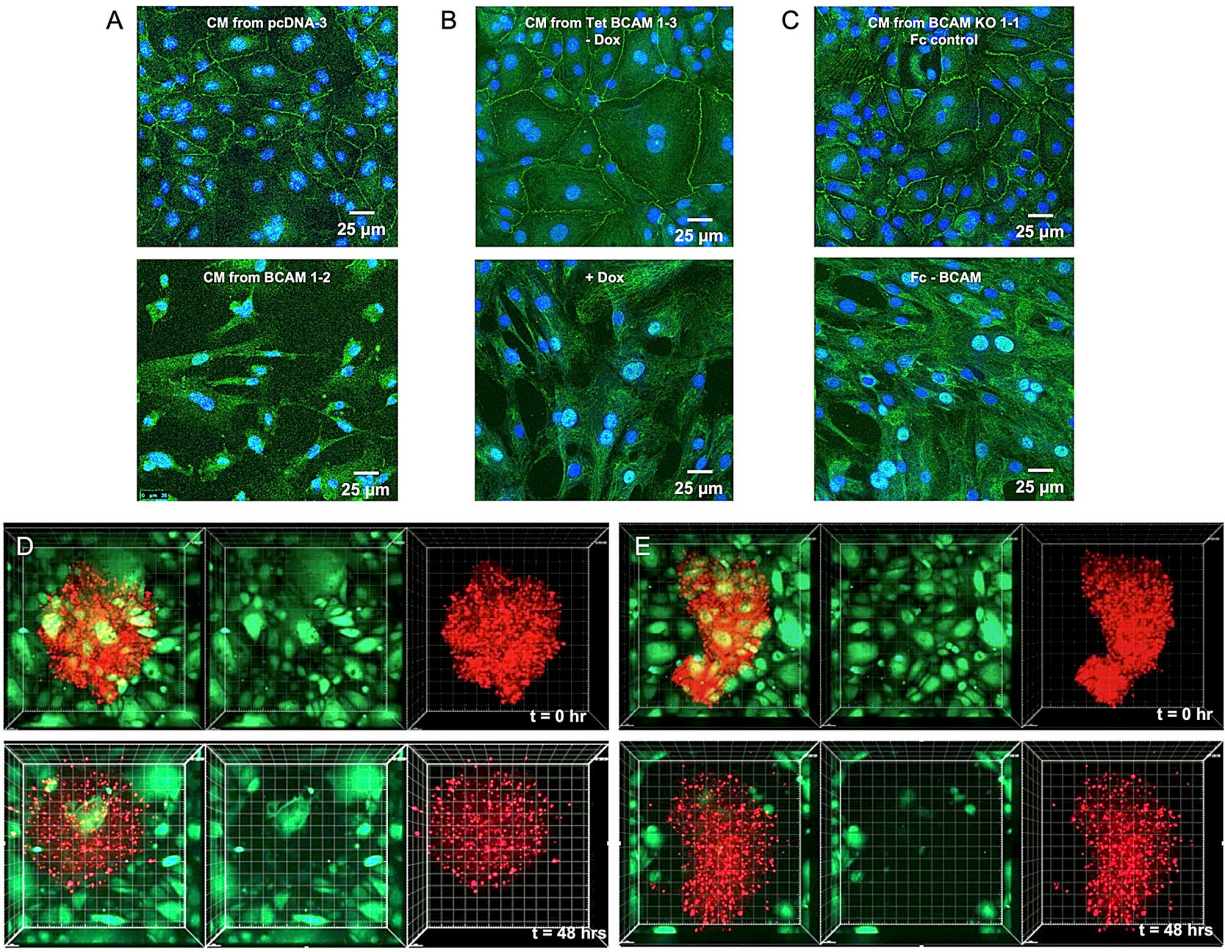
The BCAM-regulated secretome of OVCAR-8 cells induces morphological MMT and mesothelial clearance. **(A)** HPMCs exposed to CM from vector control cells (pcDNA-3) of BCAM-OE cells (BCAM1-2) for 72 h. **(B)** HPMCs treated with CM from untreated and Dox-induced TET-BCAM-1-3 cells. **(C)** HPMCs exposed to CM from BCAM-KO cells treated with Fc-BCAM (1 µg/ml) or control Fc (0.33 µg/ml) for 5 days. The tight junction marker ZO-1 was visualized by immunofluorescent staining (green). Nuclei were counterstained by Hoechst 33342 (blue). **(D**, **E)** Video microscopy images (stills from Additional files 1 and 2) showing the attachment of tumor cell spheroids (labeled with CellTracker Red) to a monolayer of HPMCs (labeled with CellTracker Green. D: vector control (pcDNA-3; panel D); E: BCAM-OE (BCAM-1-2). Images were taken at 0 and 48 h after addition of spheroids

The data presented above suggest that BCAM-induced factors may influence the differentiation state of mesothelial cells by promoting their mesenchymal transformation. This specialized form of EMT, referred to as mesothelial-to-mesenchymal transition (MMT), is critically involved in both development [55] and tumor progression [56, 57]. Immunofluorescent staining of the tight junction protein ZO-1 supports our hypothesis: HPMCs exposed to conditioned medium (CM) from BCAM-OE cells exhibited a marked redistribution of ZO-1 from intercellular junctions to the cytoplasm, accompanied by a shift from an epithelial-like morphology to an elongated, mesenchymal-like appearance (Fig. 3A and S6). Consistent results were obtained following Dox-mediated induction of BCAM (Fig. 3B and S7) or exposure of BCAM-KO cells to soluble Fc-BCAM (Fig. 3C and S8).

To study the impact of the BCAM-induced secretome in greater detail, we conducted life video recordings of tumor cell spheroids attaching to a monolayer of HPMCs. Videos 1 and 2 (Additional files 1 and 2), along with the still images in Fig. 3D and E, show a similar initial distribution of HPMCs at the attachment sites of both vector control and BCAM-OE spheroids. Subsequently, a noticeable movement of HPMCs away from BCAM-OE spheroids was observed, which was significantly less pronounced, or nearly absent, with control spheroids.

### Recombinant BCAM-induced cytokines trigger MMT

**Fig. 4:**
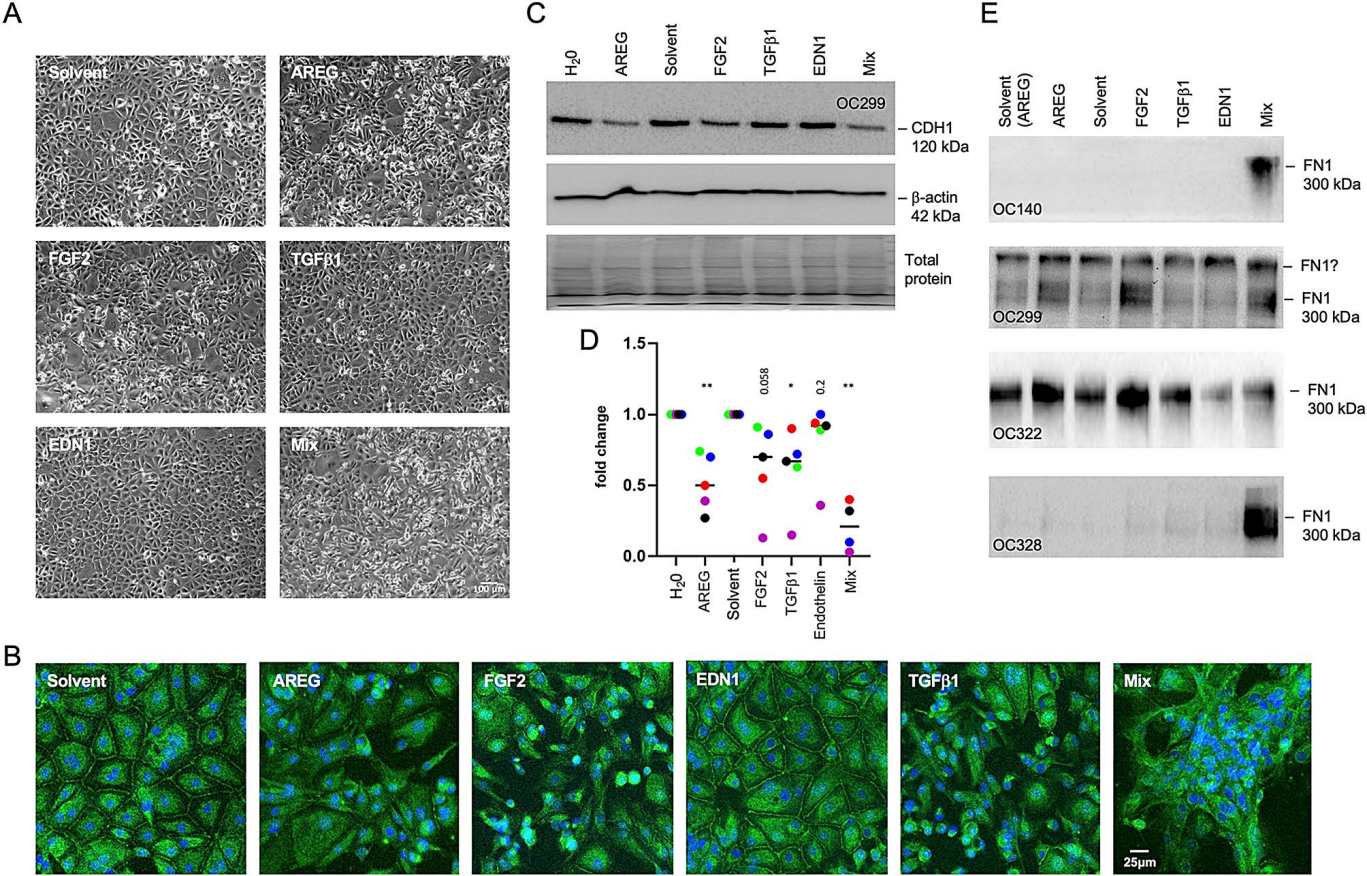
Recombinant BCAM-induced cytokines trigger MMT in HPMCs. **(A)** HPMCs were treated with recombinant AREG, FGF2, TGFβ1, EDN1 or solvent for 72 h and evaluated by phase-contrast microscopy. **(B)** ZO-1 staining (green) of HPMCs treated as in panel A. Nuclei were counterstained by Hoechst 33,342 (blue). **(C)** Immunoblot analysis of the epithelial marker CDH1 in HPMCs treated with cytokines as in panel A (representative exaµple: patient OC299). H_2_0 lane: solvent for AREG; solvent lane: solvent for all other proteins. β-actin visualization and whole protein staining were perforµed as loading controls. **(D)** Quantification of *n* = 4 5 independent experiµents as in panel B (patients OC140, OC299, OC322, OC328). *p < 0.05, **p < 0.01 by paired t test; the actual p-values are indicated for instances where the results are not statistically significant. **(E)** Immunoblot analysis of the mesenchymal marker FN1 in the same samples as in panel C. Due extreme inter-patient variability the statistical analysis of the data was inconclusive

To assess the relevance of BCAM-induced cytokines in promoting MMT, we examined the effects of recombinant AREG, FGF2, TGFβ1, and EDN1 on the morphology of HPMCs compared to solvent controls. Phase-contrast microscopy and ZO-1 staining revealed a pronounced shift towards a mesenchymal phenotype and loss of tight junctions in response to AREG and FGF2, moderate changes with TGFβ1, and no detectable effect with EDN1 (Fig. 4A and B). The most substantial mesenchymal transformation occurred when all four factors were combined (Fig. 4A). These observations were corroborated by immunoblotting, which showed a significant reduction in the epithelial marker CDH1 following treatment with AREG or FGF2 (Fig. 4C and D). In contrast, the effects of EDN1 and TGFβ1 were not statistically significant. The combined treatment with AREG, FGF2, TGFβ1 and EDN1 had a particularly strong impact (Fig. 4C and D), suggesting a synergistic effect of these cytokines within the BCAM-induced secretome.

The reduction in CDH1 expression was accompanied by a marked increase in the mesenchymal marker FN1, showing an inverse pattern to CDH1 (Fig. 4E). Although FN1 induction was observed in all four samples, the high degree of inter-patient variability prevented a conclusive statistical analysis. For instance, OC299 and OC322 exhibited high baseline levels of FN1 even without cytokine treatment, whereas OC140 and OC328 showed low to undetectable FN1 expression. In these latter samples, individual cytokines caused only weak induction, but a strong synergistic effect by the cytokine mix, not observed with OC299 and OC322 (Fig. 4E). We attribute these variations to differing levels of mesenchymal transformation in the mesothelial cells isolated from metastatic omentum, and/or variability in the number of cells undergoing MMT, thus reflecting patient-specific characteristics of the tumor microenvironment (TME).

### Transcriptional profiling indicates promotion of MMT and angiogenesis by the BCAM secretome

**Fig. 5:**
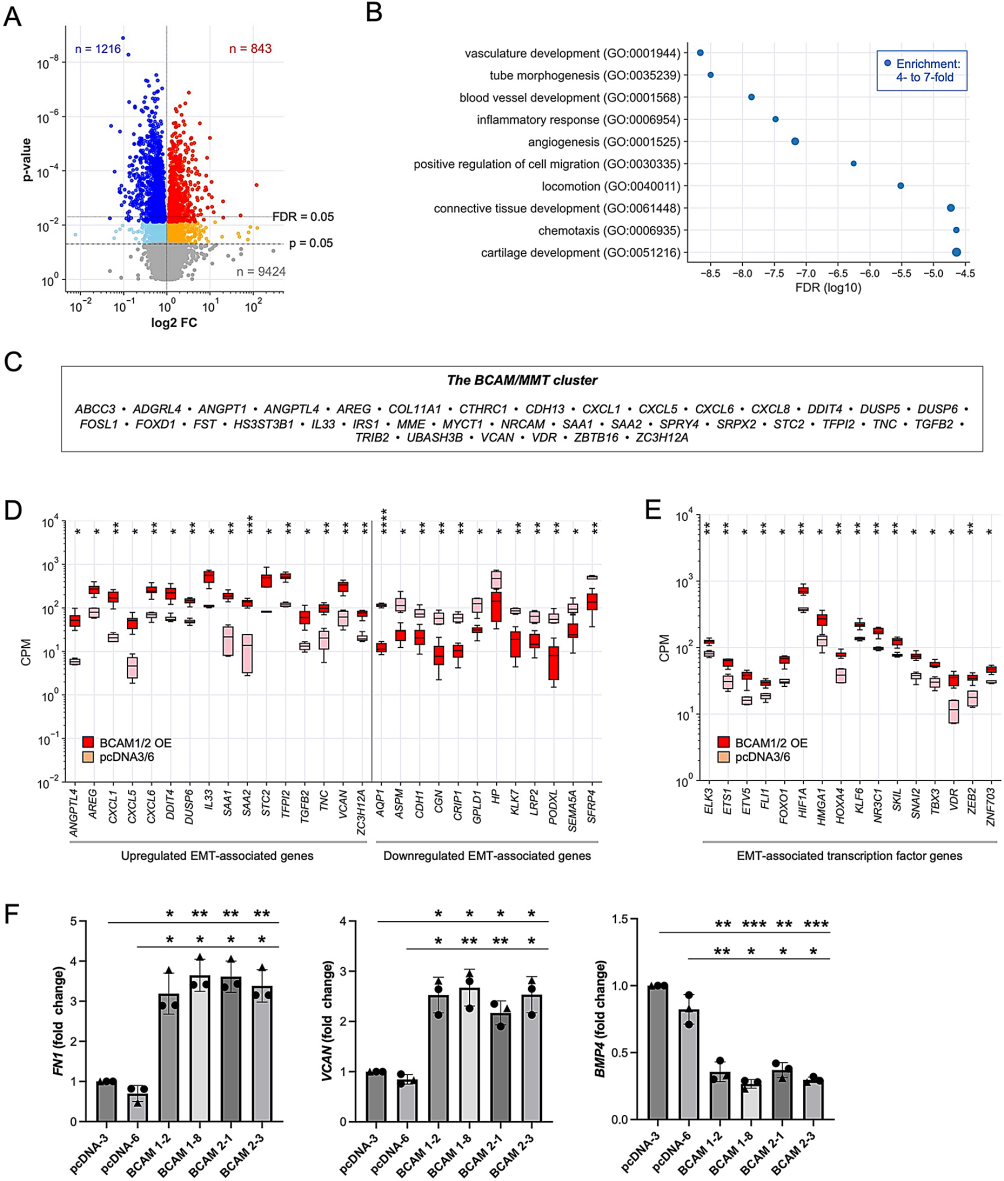
The transcriptional profile of HPMCs exposed to the BCAM secretome. **(A)** HPMCs were treated with CM from BCAM-OE cells for 72 h. The transcriptional profiles of treated and untreated were determined by RNA-Seq for *n* = 9 samples of BCAM-OE cells (clones BCAM1-2, BCAM1-8, BCAM2-3; *n* = 3 biological replicates each) and *n* = 3 samples of vector control cells (clone pcDNA-3; *n* = 3 biological replicates). The volcano plot illustrates the BCAM-induced fold change (FC) in signal intensities (log2). Blue: downregulated genes (light blue: nominal *p* < 0.05; dark blue FDR < 0.05). Orange/red: upregulated genes (orange: *p* < 0.05; red: FDR < 0.05). Grey: genes not significantly affected (*n* = 15,130). Dashed horizontal lines indicate the significance thresholds. The numbers of downregulated (blue) and upregulated (red) genes with FDR < 0.05 are shown at the top. **(B)** GO term enrichment analysis of BCAM-upregulated genes (FC > 2; FDR < 0.05; *n* = 197) using the “Biological Process Complete” function of Gene Ontology Resource [50]. The plot shows the 10 non-redundant terms with the lowest FDR. The size of the filled circles indicated the fold enrichment. **(C)** Genes of the BCAM/MMT cluster comprising *n* = 38 genes upregulated in BCAM-OE cells (FDR < 0.05; FC > 3; median CPM > 5) and previously linked to EMT. **(D)** Boxplot depicting gene expression levels (CPM) for highly expressed BCAM-regulated genes associated with EMT in the GeneCards database (median CPM > 50 in BCAM-OE cells for upregulated genes; median CPM > 50 in control cells for downregulated genes; FC > 3; FDR < 0.05). The plot shows the median (line), upper and lower quartiles (box), range (whiskers). **(E)** Boxplot as in C for BCAM-regulated genes encoding transcription factors associated with EMT (median CPM > 25; FC > 1.5; FDR < 0.05). *FDR < 0.05, **FDR < 0.01, ***FDR < 0.001; ****FDR < 0.0001 by unpaired t test and Benjamini-Hochberg adjustment in C and D. **(F)** Validation of RNA-Seq data by RT-qPCR for *FN1, VCAN* and *BMP4* expression in four BCAM-OE clones and two vector control clones (*n* = 3 biological replicates in each case). *p < 0.05, **p < 0.01; ***p < 0.001 by unpaired t test

We investigated the phenotype of HPMCs treated with conditioned media (CM) from BCAM-OE cells in further detail using RNA-Seq. This analysis identified *n* = 843 genes that were significantly upregulated (FDR < 0.05; FC > 1; median CPM > 1) in response to the BCAM-triggered secretome, while *n* = 1,216 genes showed a significant reduction (FC < 1) in expression (Fig. 5A; Table S4). GO term enrichment analysis of upregulated genes (FC > 2; FDR < 0.05; *n* = 197) revealed processes related to vasculature development, cell migration, inflammation, and connective tissue differentiation to be enriched with high significance (Fig. 5B; Table S5), aligning with the hypothesis that the BCAM secretome may promote MMT and angiogenesis. Consistent with this result, a multiple upregulated genes encode ECM components and ECM remodeling proteins (e.g., *COL1A1, COL7A1, COL1A11, COL7A13, DCN, ECM1, FN1, LOX, SERPINE1, TGFBI, THBS2, TNC* and *VCAN*) as well as EMT- and angiogenesis-inducing cytokines and growth factors, including *ANGPTL4*, *AREG, FGF2* and *TGFB2* (Table S4). Notably, several of these factors are also present in the BCAM-induced tumor cell secretome (Fig. 1C; Table S2), suggesting that BCAM initiates a pro-tumorigenic mechanism in tumor cells that is amplified by mesothelial cells.

Further supporting the induction of MMT by the BCAM secretome, *n* = 38 upregulated genes (FDR < 0.05; FC > 3; median CPM > 5; Fig. 5C; Table S6; subsequently referred to as the “BCAM/MMT cluster”) and *n* = 41 downregulated genes (FC < 0.33) have previously been associated with EMT, as indicated by entries in the GeneCards database [53, 54]. The most significantly induced and highly expressed genes within this cluster are visualized in Fig. 5D. The RNA-Seq data also revealed the induction of multiple genes coding for transcription factors linked to EMT, including the master regulators *SNAI2, ZEB1* and *ZEB2* [58] (Fig. 5E).

The regulation of *n* = 32 genes of the *n* = 38 BCAM/MMT cluster genes was corroborated by RNA-Seq analysis of HPMCs exposed to CM from Dox-induced TET-BCAM-1-3 cells, albeit with low significance (FDR > 0.05, nominal *p* < 0.05; Fig. S9; Table S7). We obtained additional evidence for MMT induction in BCAM-OE cells by RT-qPCR, showing that the mesenchymal marker genes *FN1* and *VCAN* were upregulated, while the epithelial marker gene *BMP4* was downregulated (Fig. 5F). Finally, the addition of Fc-BCAM to HPMCs did not compromise the integrity of ZO-1-containing tight junctions (Fig. S10), confirming that the induction of MMT observed above was mediated by BCAM-induced factors rather than the direct presence of BCAM in the CM.

### Expression of BCAM/MMT cluster genes in a subset of tumor-associated mesothelial cells from OC patients

To explore the expression of BCAM/MMT cluster genes in mesothelial cells within the OC TME in vivo, we reanalyzed our recently published single-cell RNA sequencing (scRNA-Seq) dataset from the metastatic omentum of three OC patients [41]. We included in this analysis cells expressing either mesothelial or fibroblast markers to account for potential MMT. Our targeted gene set contains 26 of the genes (Table S1) upregulated by the BCAM secretome according to our bulk RNA-Seq analysis. As shown in Fig. S11, these genes are coexpressed in a subset of OC-associated mesothelial cells. Notably, 2% of these cells coexpress at least 18 of the genes, while 26% showed expression of more than half of the targeted genes.

**Fig. 6:**
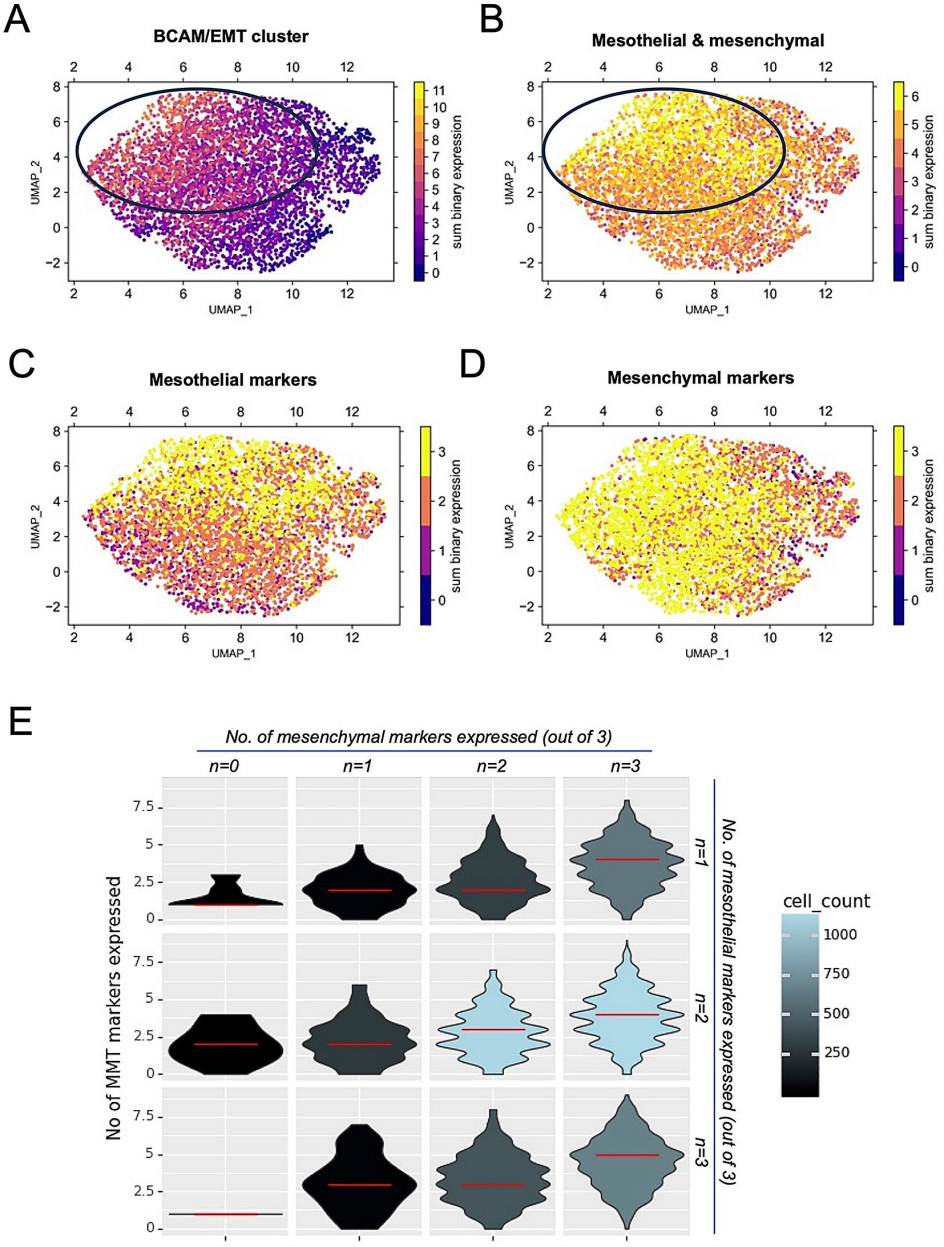
Expression of BCAM/MMT cluster genes in a subset of omental mesothelial cells from OC patients. **(A)** Expression of genes of the BCAM/MMT cluster (see Fig. 5C) in tumor-associated omental cells expressing mesothelial or fibroblast markers from three OC patients. A previously published scRNA-Seq dataset [41] was reanalyzed for expression of genes of the BCAM/MMT cluster that were covered by the targeted gene set and expressed in ≥ 100 cells (Table S1). Expression was quantified by counting the number of expressed genes in individual cells. Circled area: selective expression of BCAM/MMT cluster genes in a subset of mesothelial cells. **(B)** Quantification of the number of coexpressed mesothelial (*ITLN1, HP, UPK3B*) and mesenchymal marker genes (*DCN, FBLN1, MMP2*), which are not part of the BCAM/MMT cluster. Circled area: selective expression of these genes in a subset of mesothelial cells. **(C)** Quantification of the number of expressed mesothelial marker genes. **(D)** Quantification of the number of expressed mesenchymal marker genes. **(E)** Violin plots illustrating the co-expression of BCAM/MMT cluster genes (see Fig. 5C), mesenchymal marker genes (as in B) and mesothelial marker genes (as in B). The data show preferential expression of BCAM/MMT cluster genes in cells that coexpress at least two of the three mesenchymal markers and two of the three mesothelial markers. The color gradient represents the total number of cells in each state, with red lines indicating the median number of expressed genes within the BCAM/MMT cluster

Eleven genes are also part of the BCAM/MMT cluster defined in the preceding section (i.e., upregulated genes linked to EMT) and were analyzed in further detail (*ANGPTL4, AREG, CXCL1, CXCL6, CXCL8, FST, IL33. SAA1, SAA2, TNC and ZC3H12A*). Quantification of the number of expressed genes in individual cells revealed coexpression in a subset of mesothelial cells (circled area in Fig. 6A). We also quantified the number of expressed mesothelial (*ITLN1, HP, UPK3B*) and mesenchymal marker genes (*DCN, FBLN1, MMP2*). These genes are not part of the BCAM/MMT cluster and are therefore useful to classify cells expressing the BCAM/MMT cluster. Figure 6B shows coexpression of mesothelial and mesenchymal marker genes, Fig. 6C expression of mesothelial marker genes and Fig. 6C expression of mesenchymal marker genes. The circled area in Fig. 6B identifies a population of cells with the highest coexpression of mesothelial and mesenchymal marker genes, which coincides with cells selectively expressing genes of the BCM/MMT cluster (Fig. 6B). This association is further supported by the violin plots in Fig. 6D, showing preferential expression of genes from the BCAM/MMT cluster in cells that coexpress at least two of the three mesenchymal marker genes plus two of the three mesothelial marker genes. This pattern suggests that the BCM/MMT cluster is expressed predominantly in cells with a mixed mesothelial/mesenchymal phenotype, consistent with the promotion of MMT by the BCAM-induced secretome.

### The BCAM-regulated secretome induces endothelial tube formation

**Fig. 7:**
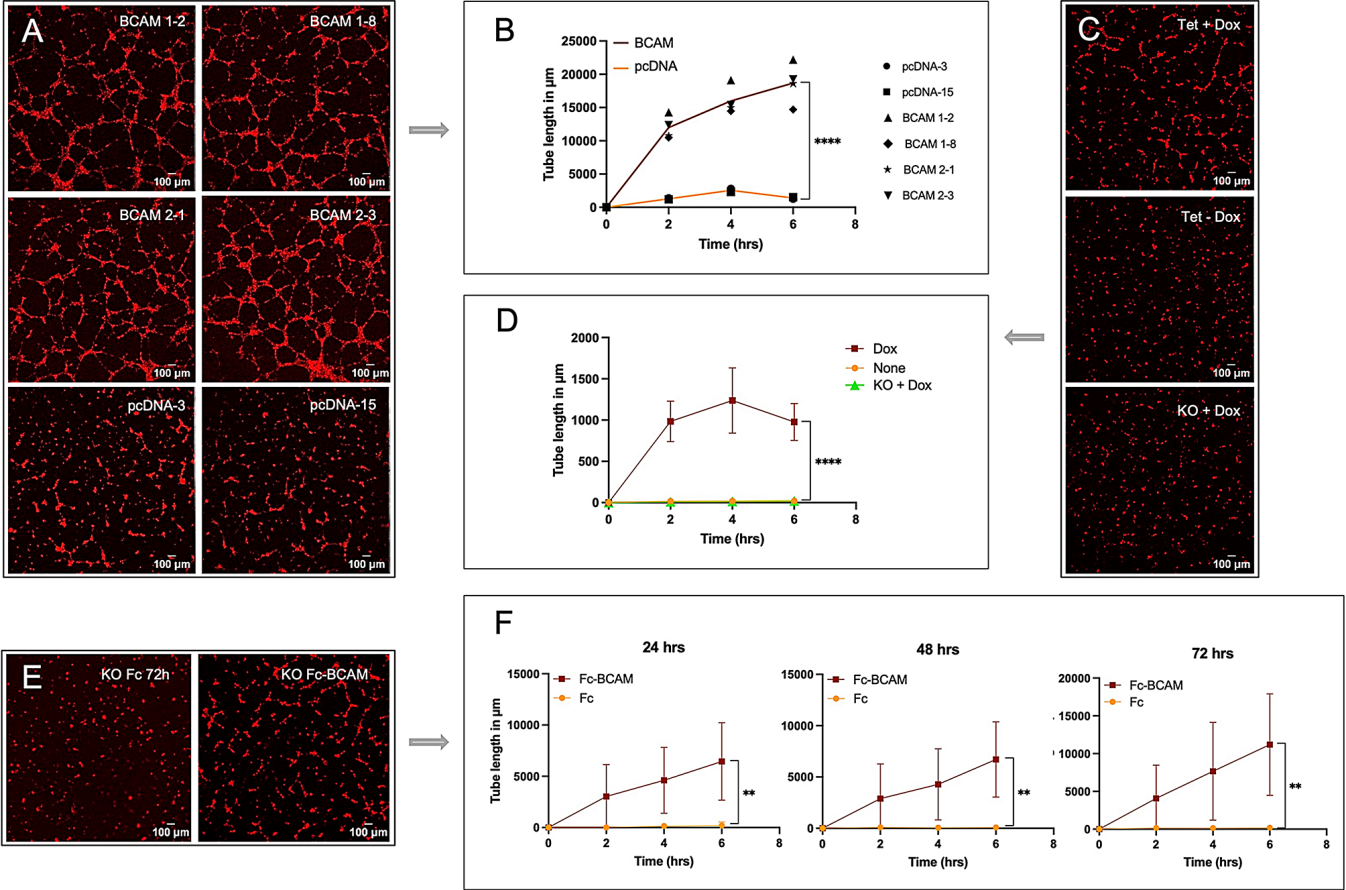
The BCAM-regulated secretome of OVCAR-8 cells induces endothelial tube formation in HUVECs. **(A)** HUVECs exposed for 6 h to CM from 4 different clones of BCAM-OE cells (BCAM1-2, BCAM1-8, BCAM2-1, BCAM2-3) or CM from 2 different vector control clones (pcDNA-3, pcDNA15) and stained with BioTracker 555 Orange cytoplasmic dye (representative example). **(B)** Quantification of *n* = 3 replicates for each clone carried out as in panel A. **(C)** HPMCs exposed for 6 h to CM from TET-BCAM-1-3 cells ± Dox (representative example). To control for potential Dox-induced off-target effects, BCAM-KO cells were treated with Dox under identical conditions. **(D)** Quantification of *n* = 3 independent experiµents carried out as in panel C. **(E)** HPMCs exposed for 6 h to CM from OVCAR8 cells treated with Fc-BCAM (1 µg/ml) or control Fc (0.33 µg/ml). **(E)** Quantification of *n* = 3 independent experiµents carried out as in panel E. **p < 0.01; ****p < 0.0001 by 2-way ANOVA in B, D and F

Besides MMT, the functional annotations in Figs. 1B and 5B revealed vascular development and angiogenesis as highly enriched biological processes induced by the BCAM-regulated tumor cell secretome (proteomics data) as well as mesothelial cells undergoing MMT (transcriptomics data). To test this hypothesis experimentally, we investigated the impact of CM from BCAM-OE cells (BCAM1-2, BCAM1-8, BCAM2-1, BCAM2-3) versus control cells (pcDNA-3, pcDNA15) in a tube formation assay using primary human endothelial cells. As shown in Fig. 7A and B, CM from all 4 BCAM-OE clones promoted tube formation with high significance. Tube formation continuously increased over a period of 6 days, and was observed irrespective of the BCAM form (BCAM1, BCAM2). Very similar results were obtained with Dox-induced TET-BCAM-1-3 cells (Fig. 7C and D). Potential off-target effects of Dox were ruled out, as no detectable impact was observed on BCAM-KO cells (Fig. 7C and D). Consistent with these findings, CM from BCAM-KO cells treated with soluble Fc-BCAM for 24, 48–72 h also induced tube formation (Fig. 7E and F).

### BCAM induces tumor angiogenesis

**Fig. 8:**
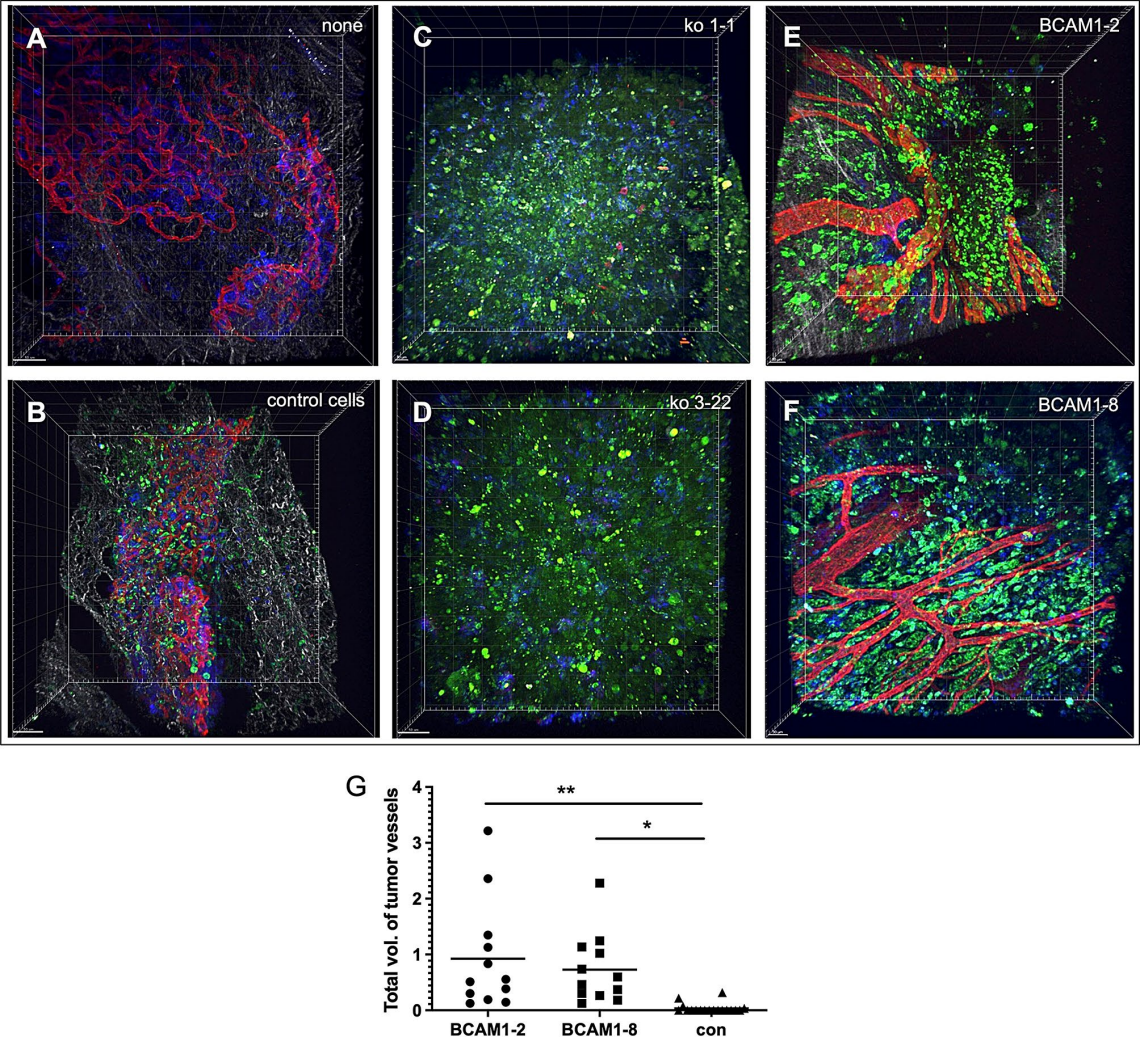
BCAM expression in OVCAR-8 cells promotes the growth of blood vessels in a mouse model of peritoneal OC dissemination. Whole-mounts of the resected omentum were stained for immune cells (CD45; blue) and blood vessels (CD31; red) and observed by multiphoton microscopy. **(A)** Representative image of the omentum from non-injected mice. **(B-F)** Omenta from mice injected i.p. with CellTracker-Green-labeled spheroids derived from the indicated cells and resected 28 days post inoculation: OVCAR-8 control cells (panel B), two different clones of OVCAR-cells with disrupted BCAM alleles (BCAM-KO-1-1, BCAM-KO-3-22; panels C and D), and or two different clones of BCAM-overexpressing OVCAR8 cells (BCAM1-2, BCAM1-8; panels E and F). The results from four additional mice are shown in Figs. S12-S15. Scale bar: 50 μm in A, B and D; 30 μm in C, E and F. **(G)** Quantification of the total volume of blood vessels (red fluorescence) in tumors from control, BCAM1-2, BCAM1-8 cells. Images were analyzed involving a machine learning-based algorithm (Imaris). Tumor volumes were normalized to 5 × 10^7^ µm^3^ to account for variations in region size. Data points (symbols) represent the mean of each image; 3–5 images per experiment (*n* = 4) were evaluated. *p < 0.05; **p < 0.01 by one-way ANOVA with Bonferroni posttest. BCAM-KO cells were not included in the quantification due to lack of tumor formation

Given the observed induction of endothelial tube formation by CM from BCAM-stimulated cells, we investigated the role of BCAM expression in OVCAR-8 cells on tumor blood vessel formation in a mouse model of peritoneal OC dissemination. To explore this, spheroids from BCAM-OE cells (two clones: BCAM1-2, BCAM1-8), from BCAM-knockout (BCAM-KO) cells (two clones: BCAM-KO-1-1, BCAM-KO-3-22), and from OVCAR-8 control cells were injected intraperitoneally into mice. After 28 days, five mice were injected with 2-NBDG to label tumor cells (green) prior to resection of the omentum and immunofluorescent staining of immune cells (CD45, blue) and blood vessels (CD31, red). The multiphoton microscopy image in Fig. 8A and S12-S15 show the normal structure of blood vessels in non-injected mice. Mice injected with OVCAR-8 control cells exhibited clearly discernible blood vessels (Fig. 8B and 12-15), which were notably reduced in tumors derived from BCAM-KO cells (Fig. 8C and D and 12-15). Conversely, a striking stimulatory effect on tumor blood vessel growth was observed in mice injected with either BCAM-OE clone (Fig. 8B and G and 12-15).

### The BCAM/MMT cluster is associated with an adverse clinical outcome in OC and multiple other carcinomas

**Fig. 9:**
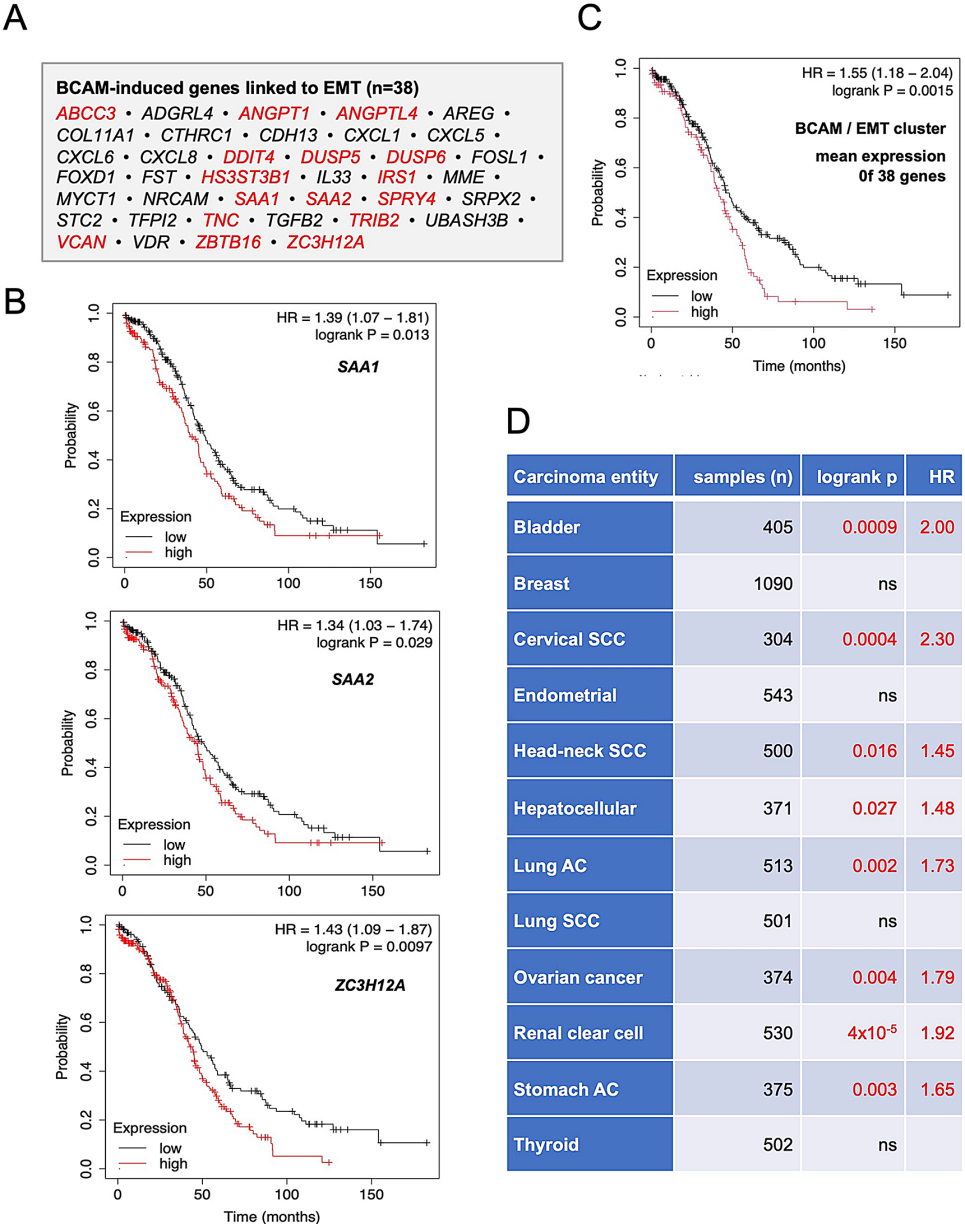
The BCAM/EMT cluster is associated with poor survival in OC and other carcinoma entities. **(A)** Genes of the 38-gene BCAM/MMT cluster, with those associated with poor overall survival highlighted in red. The analysis was performed using the Kaplan-Meier-Plotter RNA-Seq-based database [59] with auto-selected cut-offs and *n* = 373 OC patients. **(B)** Kaplan-Meier plots exemplifying the association of individual genes of the BCAM/MMT cluster with overall survival of OC. HR: hazard ratio. **(C)** Kaplan-Meier plot illustrating the association of the mean expression of all genes within the BCAM/MMT cluster with OC survival. (**D)** Pan-carcinoma overall survival association of genes of the BCAM/MMT cluster coding for secreted proteins (*n* = 16). Red: association with short survival; black: no significant association

To assess the clinical significance of the BCAM-induced secretome’s effect on mesothelial cells, we analyzed the RNA-Seq-based Kaplan-Meier-Plotter database [59] for correlations between overall survival in OC and expression of the 38 genes of the BCAM/MMT cluster identified above (Table S6). Notably, the expression of *n* = 16 genes (42.1%) within this cluster was significantly associated with poor survival outcomes (Fig. 9A; highlighted in red). This is illustrated by Kaplan-Meier survival plots for SAA1, SAA2, and ZC3H12A in Fig. 9B. Moreover, a similarly negative correlation with OC survival was observed for the cumulative mean expression of all 38 genes of the BCAM/MMT cluster (logrank *p* = 0.0015; mean hazard ratio = 1.55; Fig. 9C).

Finally, we addressed the question whether BCAM/MMT cluster genes associated with poor survival in OC might also have clinical relevance in other tumor types. This analysis was motivated by the observation that 17 of these genes encode secreted proteins, which are expressed by various cell types within the tumor microenvironment (TME), not just mesothelial cells. These proteins may therefore contribute to tumor progression irrespective of their originating cells. These genes of interest are *ANGPT1, ANGPTL4, CTHRC1, CXCL1, CXCL5, CXCL6, CXCL8, FST, NRCAM, SAA1, SAA2, SRPX2, STC2, TFPI2, TGFB2, TNC* and *VCAN*. In OC, for example, most of these genes are highly expressed by carcinoma-associated fibroblasts (CAFs) [36], which are crucial components within the TME of most cancers [60]. We, therefore, analyzed survival associations for these genes across 12 carcinoma types, each with at least 300 patients in the Kaplan-Meier-Plotter database. Remarkably, significant associations with shorter overall survival were observed for 75% (8 out of 12) of the carcinoma types analyzed, including lung, ovarian and stomach adenocarcinomas (highlighted in red in Fig. 9D; log-rank p: 0.00004–0.04; mean HR: 1.33–3.26). These findings suggest that the clinical relevance of the BCAM/MMT cluster extends beyond OC.

## Discussion

Although BCAM is abundant in the OC TME and associated with shorter relapse-free survival [4], its pro-metastatic functions remain only partially understood. Similar to its role in other cancers [13–17]. BCAM influences tumor cell adhesion and migration; however, in OC its effect appears to be inhibitory, suggesting a potential tumor-suppressive role [7]. Nevertheless, in the same study, we uncovered novel functions of BCAM in OC that may contribute to metastasis by promoting the trans-mesothelial invasion of tumor cells into peritoneal organs, particularly the omentum. Thus, BCAM was found to facilitate the dispersion of tumor cell spheroids within a 3D matrix by inhibiting spheroid compaction, and to induce the clearance of mesothelial cells at sites of spheroid attachment, a critical step in the trans-mesothelial invasion of tumor cells. Despite the potential significance of this effect in metastasis formation, the underlying mechanisms remained unclear. Likewise, other functions of BCAM that might promote OC progression had not been identified. The present study, therefore, specifically addressed these questions.

**Fig. 10:**
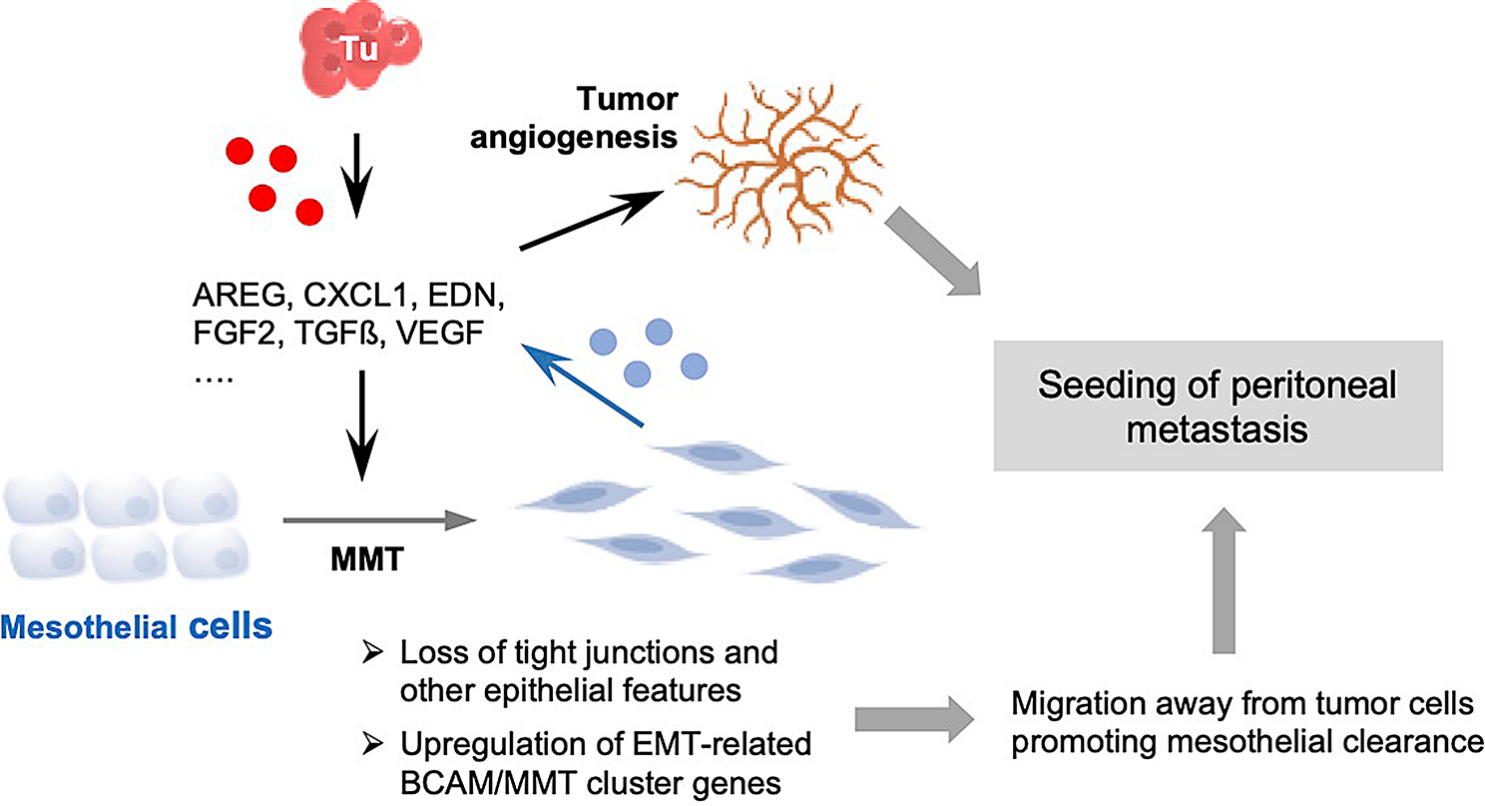
Model illustrating potential functions of BCAM in MMT and tumor angiogenesis. BCAM expression by OC cells (Tu) drives the secretion of cytokines (red dots), which facilitate MMT and promote tumor angiogenesis. Salient features of MMT are an elongated cellular morphology, disruption of tight junctions, loss of other epithelial features, and the upregulation of an EMT-associated gene cluster (BCAM/MMT cluster). This mesenchymal transition triggers mesothelial cell migration (repulsion) from tumor attachment sites, resulting in the clearance of the mesothelial layer, sub-mesothelial tumor cell invasion, and subsequent metastatic growth. Furthermore, the secretome released by mesothelial cells undergoing MMT (grey dots) amplifies both MMT and tumor angiogenesis, creating a self-reinforcing cycle

Our work identified MMT as the primary BCAM-driven mechanism that promotes mesothelial clearance through secretion of cytokines and growth factors (see model in Fig. 10). These factors were identified via affinity proteomics and/or ELISA in conjunction with the information in the GeneCards database [53, 54]. A causative role of BCAM is strongly supported by the consistency of results obtained with CM of BCAM-overexpressing (BCAM-OE) cells, CM from OVCAR8 cells with doxycycline-inducible BCAM, and CM from BCAM-knockout (BCAM-KO) cells exposed to soluble Fc-BCAM. BCAM-induced proteins, previously reported to drive EMT and associated processes such as cell migration in various cancers, include AREG [61], CXCL1 [62], CXCL10 [63], EDN1 [64], FGF2 [65], TGFβ1 [66] and VEGFB [67]. We confirmed MMT-promoting effects on HPMCs exemplarily for AREG, FGF2, and TGFβ1. These findings collectively provide robust evidence that BCAM induces the secretion of multiple cytokines and growth factors and thus promotes MMT.

In line with observations of EMT/MMT in other experimental systems [68], the BCAM secretome triggered a loss of intercellular contacts through reorganization of ZO-1-containing tight junctions. The ensuing disruption of mesothelial monolayer integrity with the occurrence of intercellular gaps likely facilitates the attachment of tumor cells to the underlying ECM and thus the trans-mesothelial invasion into sub-mesothelial structures in vivo [64]. This mechanism has previously been suggested for the introduction of mesothelial gaps by tumor-cell-exerted mechanical force [18]. Additionally, BCAM-induced MMT enhanced another key characteristic of EMT, i.e., cell motility [68]. This increased motility unexpectedly manifested as mesothelial cell migration away from the spheroid attachment site, suggesting a repulsive effect of BCAM-expressing tumor cells on mesothelial cells.

Secreted proteins that mediate cell repulsion are known to play critical roles in various developmental processes, including axon guidance, endothelial cell migration, lung branching, and immune cell activation [69]. Among these, members of the semaphorin (SEMA) and ephrin (EPH) families are particularly significant [69–72]. Notably, our affinity proteomics data indicate a strong induction of several SEMA and EPH family proteins in BCAM-OE cells, particularly SEMA4C, EFNA1, EFNA4 and EFNB2. This raises the intriguing hypothesis that BCAM-induced mediators of MMT may cooperate with mesothelial-cell-repulsive proteins to clear metastatic target sites. It is conceivable that this mechanism may contribute to the absence of a mesothelial layer in micrometastatic areas, as observed in histological studies [7, 18].

EMT has also been linked to a phenomenon known as contact inhibition of locomotion [73, 74], where cells move away from each other following cell-cell contact. This process is typically mediated by a switch in cadherin types, such as from E-cadherin (encoded by the CDH1 gene) to N-cadherin (encoded by the CDH2 gene) [75]. Our RNA-Seq data reveal a significant reduction in *CDH1* expression associated with BCAM-triggered MMT, but no notable changes in *CDH2*. However, we observed a marked increase in *CDH20* expression. While CDH20 is known to play a critical role in cancer cell migration [76], its involvement in cell repulsion has not yet been reported. Thus, the role of contact inhibition of locomotion and cadherin switching in the context of our findings remains the subject of future studies.

Transcriptional profiling confirmed that mesothelial cells undergo a mesenchymal transition in response to the BCAM secretome. This was evident from the upregulation of numerous structural components of ECM, ECM-modifying enzymes and transcription factors that drive EMT. Additionally, the BCAM secretome upregulated of multiple cytokines and growth factors known for their pro-metastatic roles in OC, including ANGPTL4 [77], AREG [78], CXCL8 [79], FGF2 [80], IL6 [81], IL33 [82], STC1 [77], TGFB2 [83] and WNT5A [84]. These factors promote, for example, cancer cell invasion, angiogenesis, immunosuppression and/or chemoresistance, which may partially be related to EMT/MMT.

In vitro and in vivo models provided direct experimental evidence that the BCAM-induced secretome of OC cells promotes blood vessel formation. Thus, endothelial tube formation in vitro was enhanced in response to overexpression of BCAM1 or BCAM2, Dox-mediated induction of BCAM1 or exposure to soluble Fc-BCAM. This was further validated in a mouse model of peritoneal dissemination for BCAM-OE cells, where a dramatic BCAM-dependent increase in tumor blood vessel formation was observed. The secreted factors induced by BCAM in tumor cells, and indirectly through the tumor cell secretome in mesothelial cells, likely cooperate to promote angiogenesis, e.g. through VEGFB [85] produced by tumor cells, CXCXL8 [86] expressed by mesothelial cells and FGF2 [87] secreted by both cell types. Cooperation of tumor and mesothelial cells may also amplify the induction of MMT, as several relevant factors in the BCAM-induced tumor cells secretome were also transcriptionally upregulated in mesothelial cells undergoing BCAM-induced MMT. These observations are consistent with findings from other experimental systems suggesting that mesothelial cells are a crucial source of CAFs generated through MMT, enhancing the peritoneum’s susceptibility to cancer cell invasion and facilitating secondary tumor growth by promoting angiogenesis [56].

Multiple lines of evidence indicate that these findings may have clinical significance:

(i) Disrupted mesothelial layers are characteristic of peritoneal metastatic sites in OC and gastrointestinal cancers spreading by transcoelomic dissemination [7, 18, 88, 89, 90];
(ii) myofibroblasts expressing mesothelial markers are found at metastatic sites in patients with peritoneal dissemination [56];
(iii) the concentration of soluble BCAM correlates with the level of BCAM-induced MMT- and angiogenesis-promoting cytokines in the ascites of OC patients;
(iv) scRNA-Seq data revealed coexpression of genes of the BCAM/MMT cluster in a subpopulation of tumor-associated mesothelial cells from OC patients;
(v) the majority of these genes is associated with a shorter overall survival of OC; and.
(vi) expression of the BCAM/MMT cluster is also linked to a poor clinical outcome of other carcinomas.

The induction of cytokines and growth factors appears to be central to the BCAM-mediated effects observed in this study. However, the precise mechanisms by which BCAM triggers the secretion of these factors to promote MMT and angiogenesis remain unclear. Notably, full-length BCAM1, a truncated form of BCAM2 lacking most of the putative intracellular signaling domain, as well as soluble Fc-BCAM all exhibited similar effects. This suggests that BCAM does not act as a conventional receptor in this context but rather functions as a ligand or inhibitory decoy, akin to its role in disrupting the interaction between LAMA5 and integrin β1 [7, 13]. However, our data indicate that the induction of cytokines by BCAM is independent of an LN-511 matrix and unaffected by an activating antibody for integrin β1, pointing to a novel mode of action, as all other reported interactions of BCAM, i.e., with α3β1, α4β1, α6β1 or α7β1, involve integrin β1 [13, 22, 23]. Identifying the molecules involved in this process, such as discovering an unknown BCAM receptor or elucidating BCAM-induced changes in signal transduction through unbiased phosphoproteomics, represents an intriguing avenue for future research.

## Conclusion

We have discovered two previously unrecognized functions of the BCAM-induced secretome that may play roles at different stages of transcoelomic metastasis: BCAM’s influence on MMT could promote the formation of micrometastases at peritoneal attachment sites, while neo-angiogenesis is crucial for tumor expansion. The potential clinical significance of these findings highlights BCAM as an intriguing candidate for improved therapeutic strategies. Given that BCAM likely functions as a ligand or decoy, either expressed on the cell surface or shed in a soluble form, there is a possibility of developing blocking agents that inhibit crucial BCAM interactions. Once suitable candidates are identified, this approach could be readily tested in follow-up studies using the in vitro assays and mouse model described in this manuscript. Additionally, BCAM may represent a promising target for the development of diagnostic tools, which could help in selecting patients for tailored therapies, a hypothesis that warrants further investigation in future studies.

## Electronic supplementary material

Below is the link to the electronic supplementary material.

Supplementary Material 1: Video 1 - pcDNA6

Supplementary Material 2: Video 2 - BCAM1-2

Supplementary Material 3: Supplementary Figures S1-S15

Supplementary Material 4: Supplementary Tables S1-S7

## Data Availability

RNA-seq data were deposited at EBI Array Express under accession numbers E-MTAB-14411 and E-MTAB-14412. All other data generated or analyzed in this study are included in the supplementary files or have been deposited as part of previous publications cited in the text.

## Abbreviations

BCAM: Basal cell adhesion molecule
BCAM-OE cells: BCAM-overexpessing cells
CM: Conditioned medium
ELISA: Enzyme-linked immunosorbent assay
FDR: False discovery rate
HPMC: Human peritoneal mesothelial cell
MFI: Mean fluorescence intensity
LN-511: Laminin-511 (α5/β1/γ1-laminin)
LAMA5: Laminin α5
KO: Genetic disruption (“knockout”)
NPX: Normalized protein expression
OC: Ovarian cancer
RT-qPCR: Quantitative reverse transcription polymerase chain reaction
scRNA-Seq: Single-cell RNA sequencing
RNA-Seq: RNA sequencing
RFS: Relapse-free survival
TME: Tumor microenvironment
