# Supplementary material for: Basal cell adhesion molecule (BCAM) promotes mesothelial-to-mesenchymal transition and tumor angiogenesis through paracrine signaling
Source: Cell Commun Signal. 2025 Mar 13;23:136. doi: 10.1186/s12964-025-02128-9 (PMC11907816; doi:10.1186/s12964-025-02128-9)
Supplement: Supplementary file 3 — Supplementary Material 3: Supplementary Figures S1-S15 [file 12964_2025_2128_MOESM3_ESM.pdf]

SUPPLEMENTAL FIGURES

Basal cell adhesion molecule (BCAM) promotes mesothelial-to-mesenchymal transition and tumor angiogenesis through paracrine signaling

Suresh Sivakumar[1], Sonja Lieber[¶1,2] Raimund Dietze[¶1], Vanessa M. Beutgen[3,4], Eileen C. Sutor[1], Sophie Heidemann[1], Florian Finkernagel[1,5], Julia Teply-Szymanski[5], Andrea Nist[5], Thorsten Stiewe[5,6,7], Katrin Roth[8], Silke Reinartz[1], Johannes Graumann[3,4], Sabine Müller-Brüsselbach[1] and Rolf Müller*[1]

[1]Translational Oncology, Center for Tumor Biology and Immunology (ZTI), Philipps University, Marburg, Germany
[2]Institute of Systems Immunology, Center for Tumor Biology and Immunology (ZTI), Philipps University, Marburg, Germany
[3]Institute of Translational Proteomics, Biochemical/Pharmacological Centre, Philipps University, Marburg, Germany
[4]Core Facility Translational Proteomics, Philipps University, Marburg, Germany
[5]Genomics Core Facility, Philipps University, Marburg, Germany
[6]Institute of Molecular Oncology, Member of the German Center for Lung Research (DZL), Philipps University, Marburg, Germany
[7]Institute of Lung Health, Justus-Liebig University, Giessen, Germany
[8]Cell Imaging Core Facility, Center for Tumor Biology and Immunology (ZTI), Philipps University, Marburg, Germany

¶S. Lieber and R. Dietze contributed equally to this work.
*Correspondence: rolf.mueller@uni-marburg.de

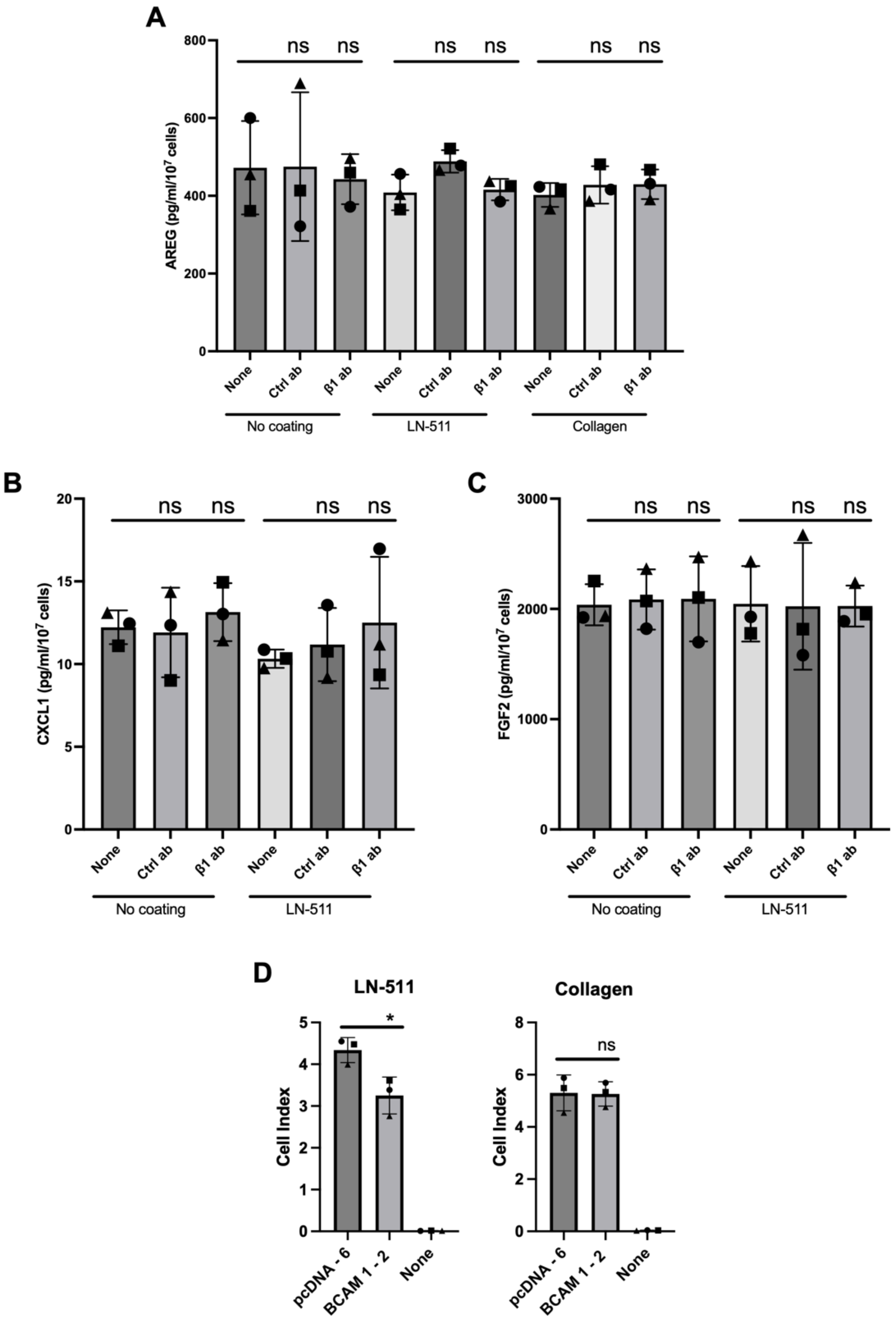

Figure S1: Role of LN-511 and integrin β1 in BCAM-induced secretion of cytokines and cell adhesion. (A-C) BCAM1-2 cells were plated on uncoated or LN511-coated-dishes, as well as in the presence of an integrin-β1-activating (β1 ab) antibody or a negative control anitbody (Ctrl ab). In panel A cells were additionally plated on collagen. CM was collected 48 hrs after medium change, and the concentrations of AREG (A), CXCL1 (B) and FGF2 (C) were quantified by ELISA (n=3 biological replicates). ns, not significant by paired t test. **(D)** Effect of BCAM on OC cell adhesion to LN-511 on non-adhesive microplates coated with LN-511. Cell adhesion was quantified by xCELLigence real-time cell analysis (RTCA). The plot is based on our previously published data (ref. 7) for n=3 biological replicates. *$p<0.05$; ns, not significant by unpaired t test.

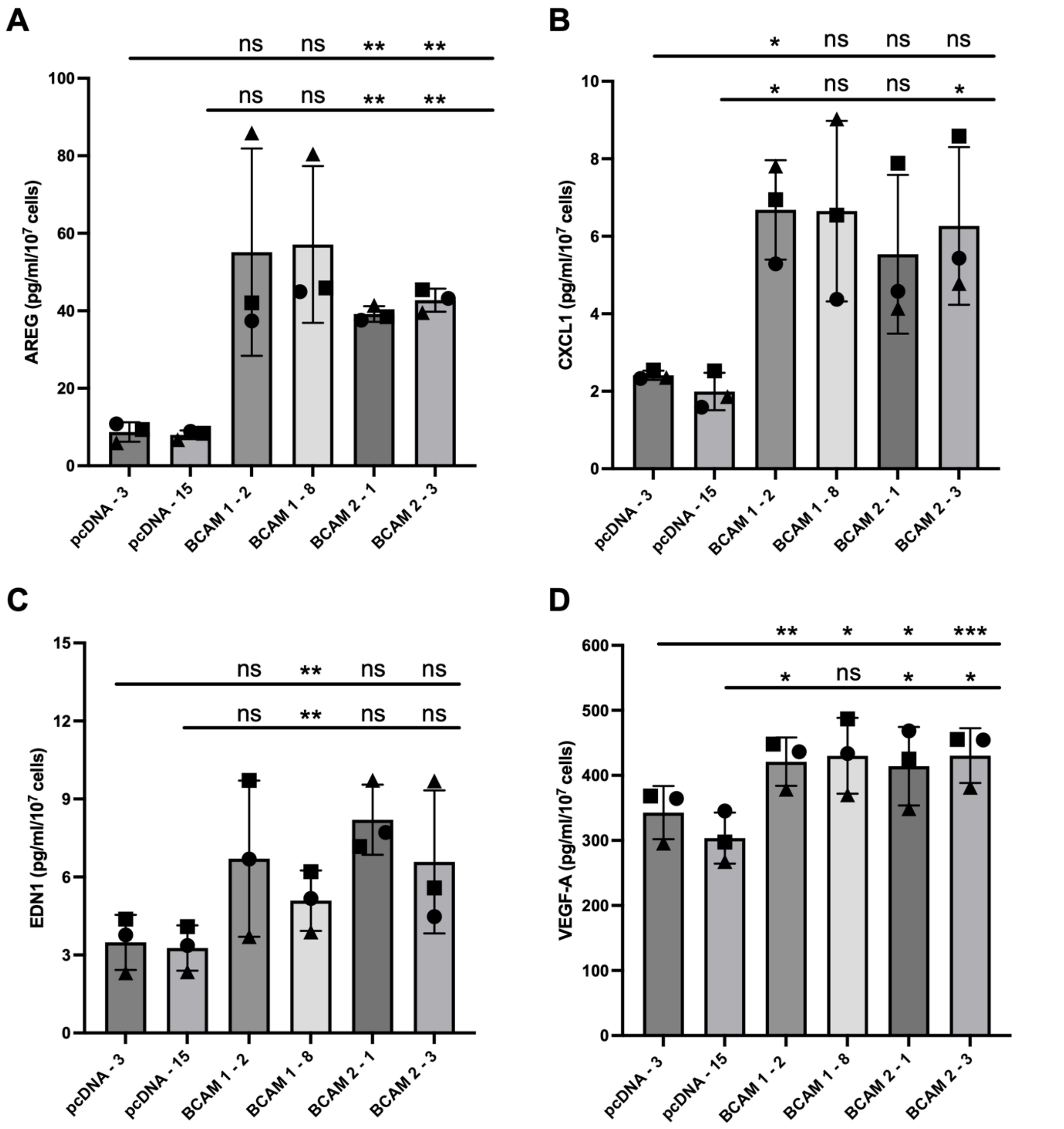

Figure S2: Verification of PEA-based affinity proteomics data by ELISA. (A, B) Comparative analysis of CM from 4 different BCAM-OE clones (BCAM1-2, BCAM1-8, BCAM2-1, BCAM2-3) and two vector control clones (pcDNA-3, pcDNA-15) as in Fig. 2A for AREG (A), CXCL1 (B), EDN1 (C) and VEGFA (D). n=3 biological replicates were analyzed for each clone. *$p<0.05$, **$p<0.01$, ***$p<0.001$ by unpaired t test. ns, not significant.

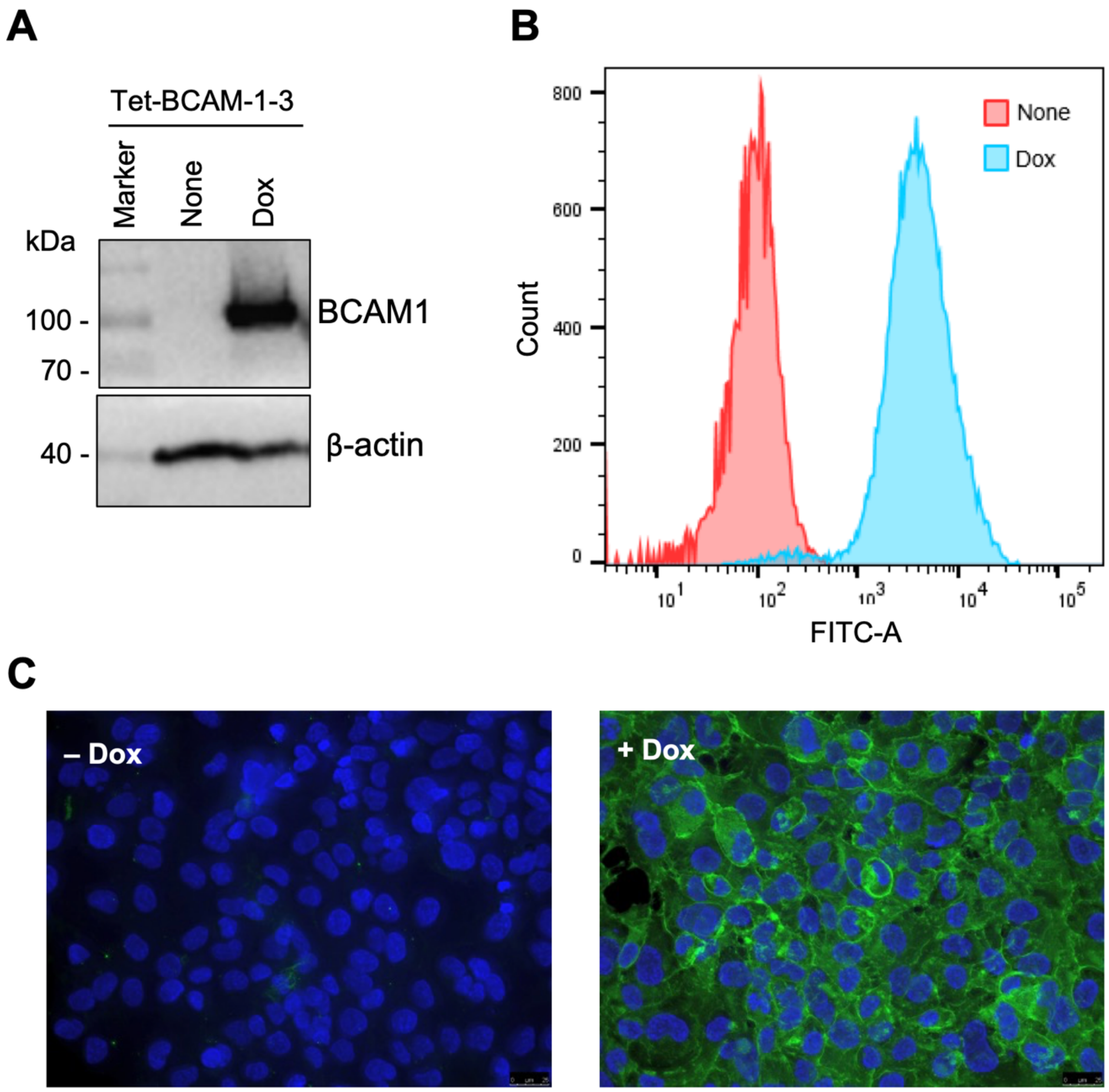

Figure S3: Characterization of TET-BCAM-1-3 cells. (A) Immunoblot of TET-BCAM-1-3 cells incubate in the presence or absence of Dox for 48 hrs. (B) Flow cytometry of BCAM surface expression. The histogram shows of TET-BCAM-1-3 cells ±Dox stained with a BCAM-specific antibody. **(C)** Immunofluorescent staining of cells TET-BCAM-1-3 cells ±Dox. Green: BCAM-specific antibody; blue: Hoechst 33342 .

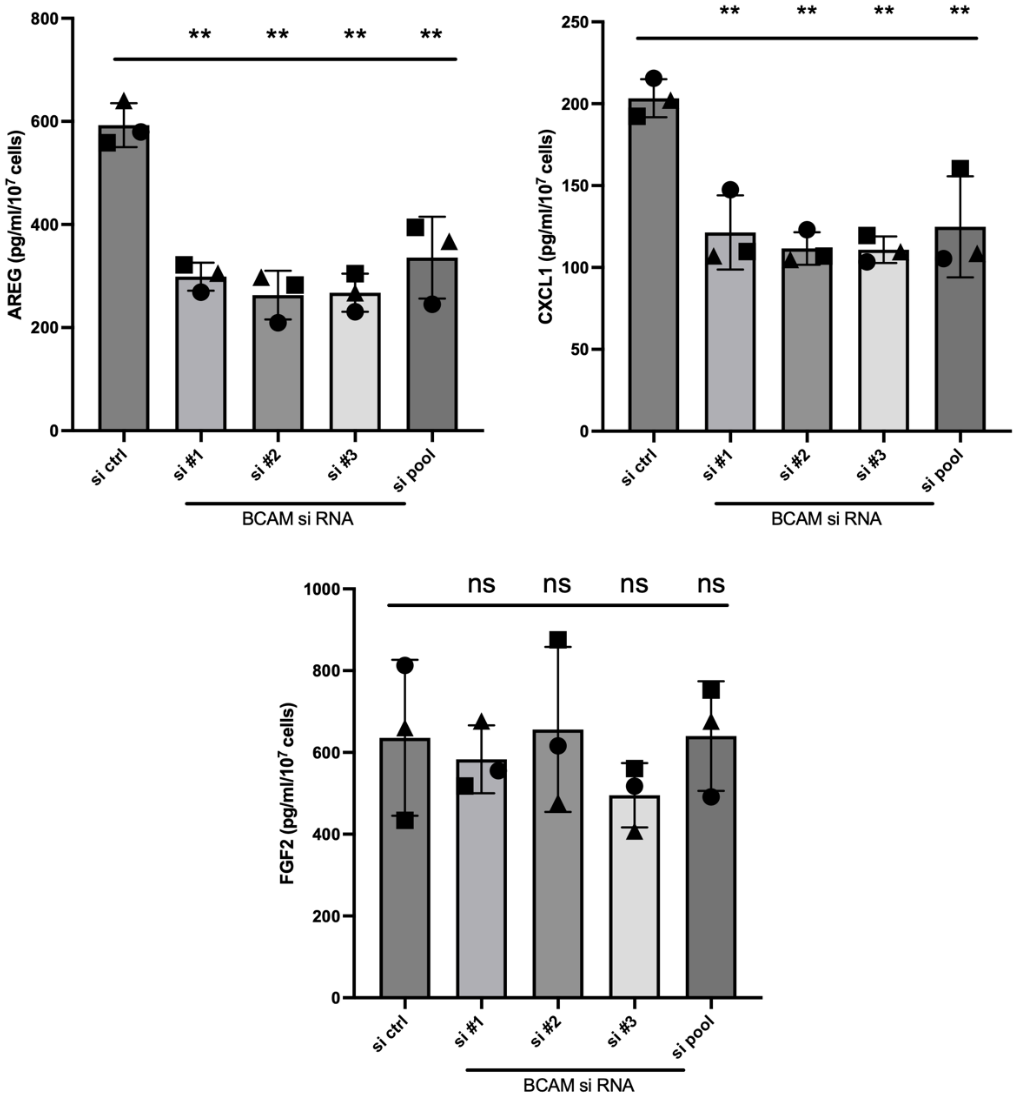

Figure S4: siRNA-mediated inhibition of the BCAM-induced secretion of cytokines and growth factors. CM from OVCAR4 cells was collected 48 hrs after medium change, and the concentrations of AREG, CXCL1 and FGF2 were quantified by ELISA (n=3 biological replicates). **p<0.01 by unpaired t test; ns, not significant.

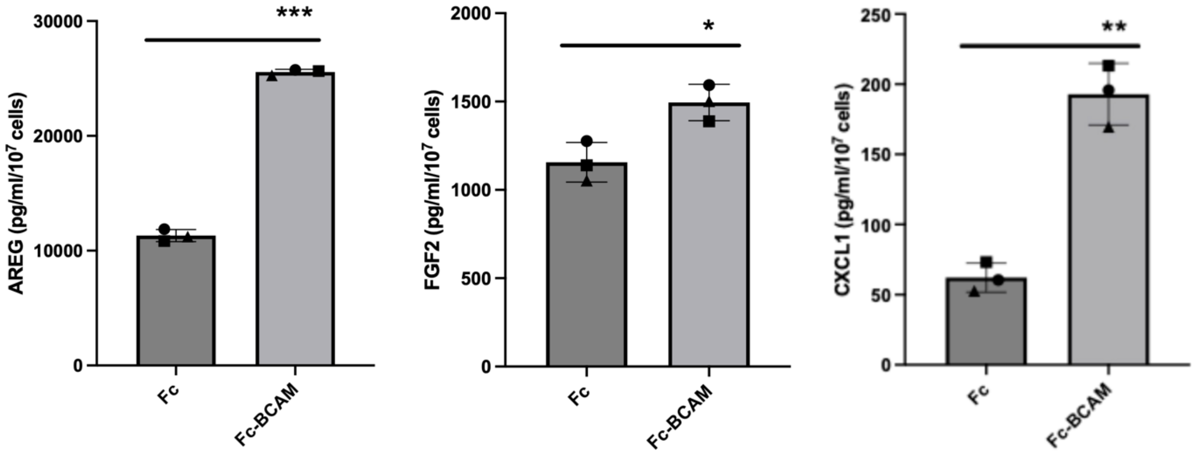

Figure S5: BCAM-induced secretion of cytokines and growth factors in patient-derived OC cells. CM from OCMI-91s cells was collected 72 hrs after medium change, and the concentrations of AREG, CXCL1 and FGF2 were quantified by ELISA (n=3 biological replicates). *p<0.05, **p<0.01, ***p<0.001 by paired t test; ns, not significant.

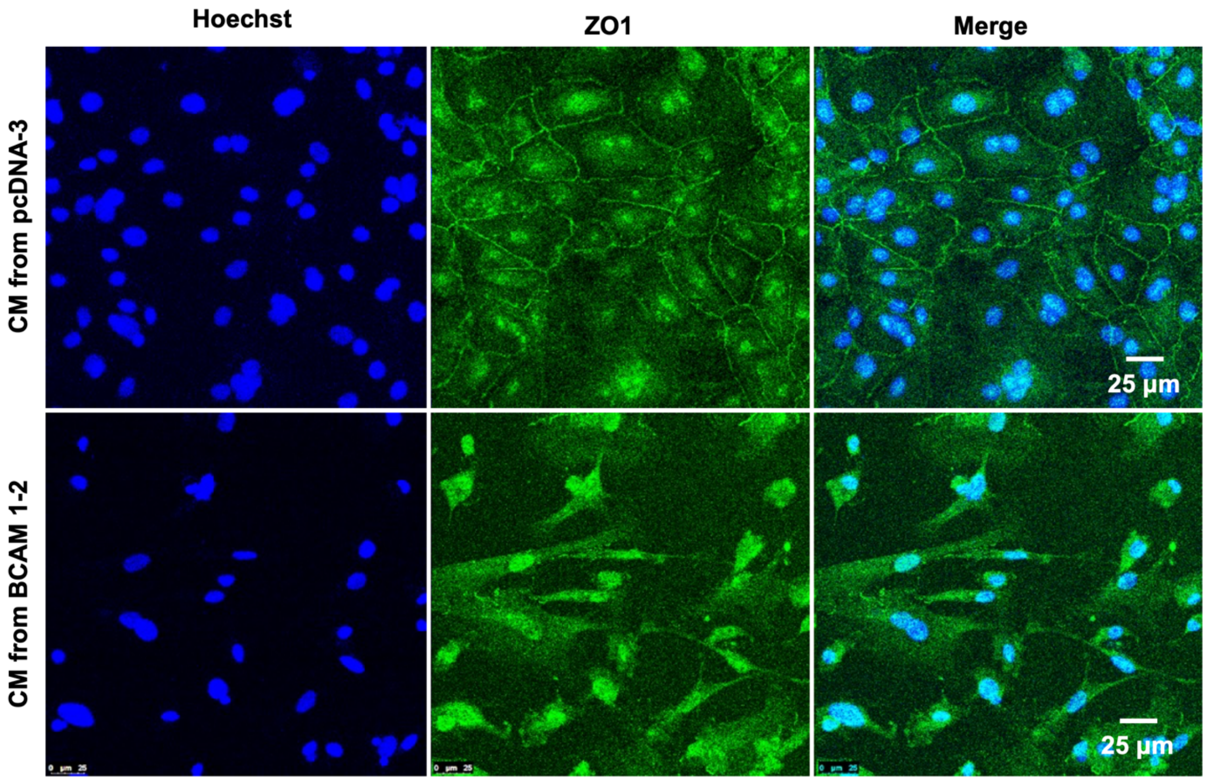

Figure S6: Induction of morphological MMT in BCAM-OE cells. HPMCs were exposed to CM from pcDNA-3 or BCAM1-2 cells for 72 hrs, and ZO-1 was visualized by immunofluorescent staining (green). Nuclei were counterstained by Hoechst 33342 (blue). The merged image is identical to Fig. 3A.

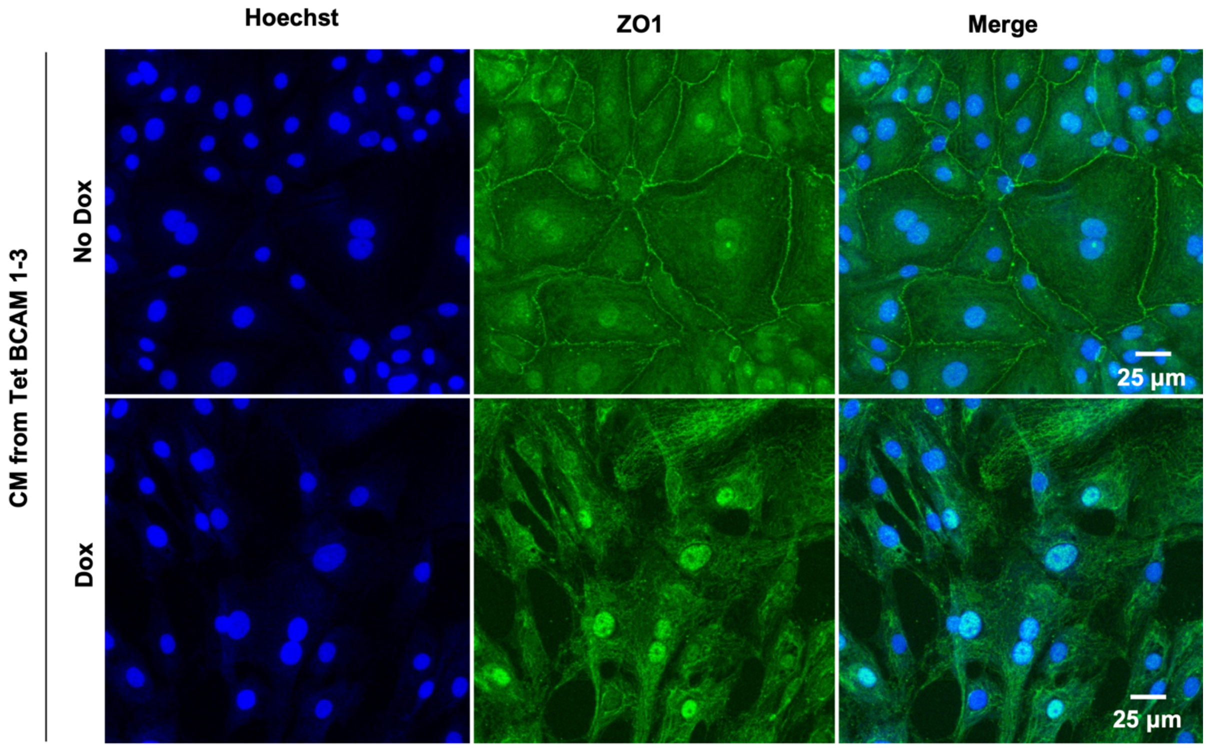

Figure S7: Inducible induction of morphological MMT in TET-BCAM-1-3 cells. HPMCs were exposed to CM from TET-BCAM-1-3 ±Dox for 72 hrs and stained as in Fig. S6. The merged image is identical to Fig. 3B.

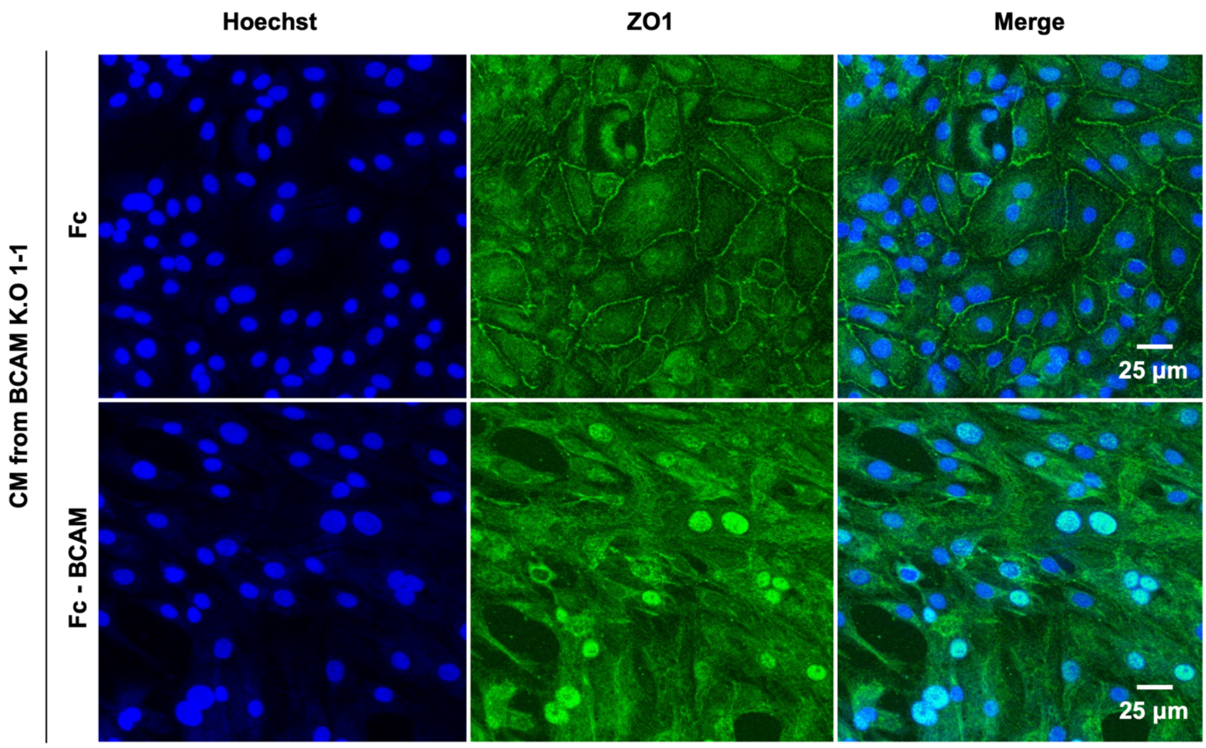

Figure S8: Induction of morphological MMT by soluble Fc-BCA. HPMCs were exposed to CM from BCAM-KO cells treated with Fc-BCAM or control Fc for 3 days. and stained as in Fig. S7. The merged image is identical to Fig. 3C.

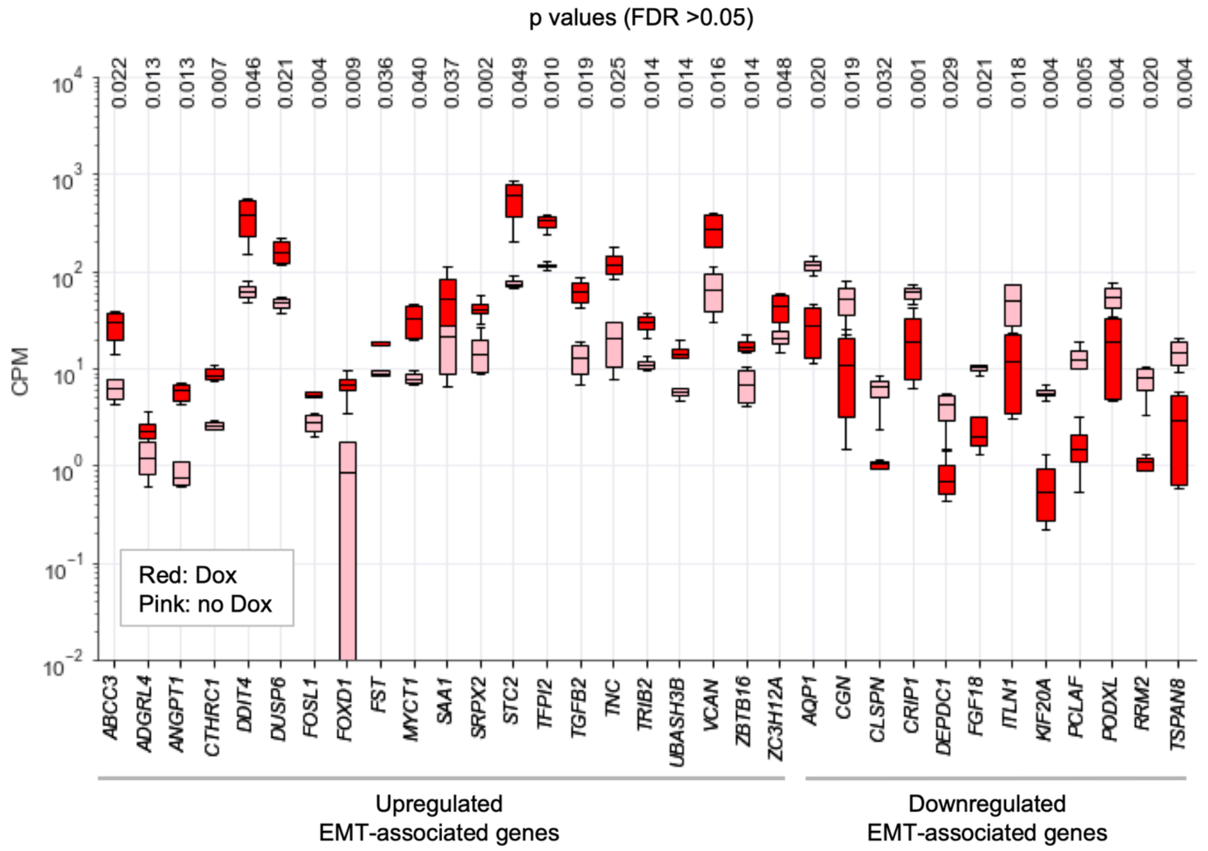

Figure S9: Induction of EMT-associated genes in TET-BCAM-1-3 cells. HPMCs were exposed to CM from Dox-induced (12 days) or non-induced TET-BCAM-1-3 cells and analyzed by RNA-Seq. The boxplot depicts gene expression levels (CPM) for BCAM-regulated genes associated with EMT in The GeneCards database (as in Fig. 5C). The plot shows the median (line), upper and lower quartiles (box), range (whiskers) for n=4 biological replicates. As all FDRs were above the threshold of >0.05 the nominal p values are shown at the top.

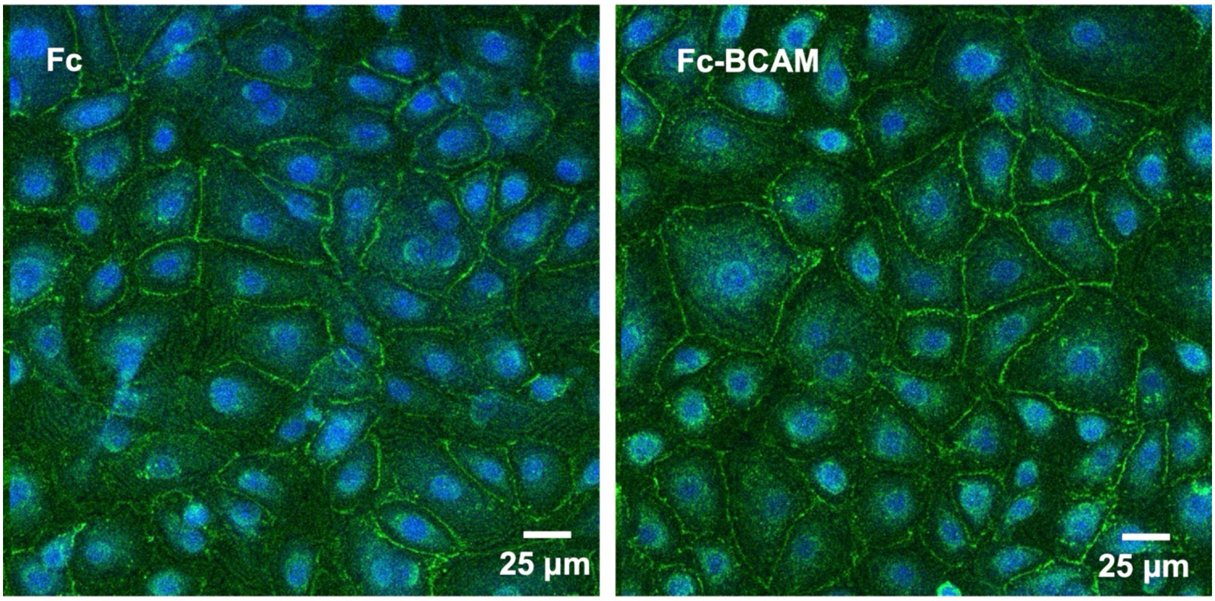

Figure S10: Integrity of ZO-1-containing tight junctions in HPMCs following Fc-BCAM treatment. Fc-BCAM (1 µg/ml) or control Fc (0.33 µg/ml; equimolar to Fc-BCAM) was added to HPMCs at 0, 24 and 48 hrs. Ater another 24-hour incubation the cells were analyzed, corresponding to a total exposure time of 72 hrs. The tight junction marker ZO-1 was visualized by immunofluorescent staining (green). Nuclei were counterstained by Hoechst 33342 (blue). The images show no discernible difference between both treatments. This contrasts with the treatment using conditioned medium from BCAM-OE cells, which clearly induced MMT after 72 hrs of incubation. The concentration of Fc-BCAM used in the experiment shown exceeds the maximum level of soluble BCAM found in OS ascites i.e., 520 ng/ml (median 76 ng/ml) [7].

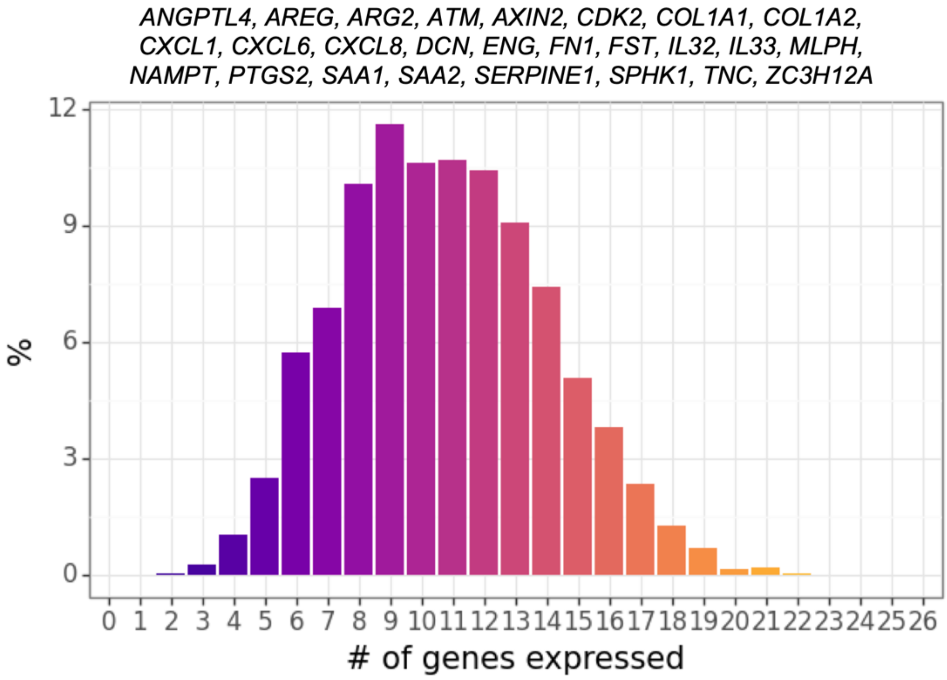

Figure S11: Coexpression of genes induced by the BCAM secretome in a subset of omental mesothelial cells from OC patients. Our published scRNA-Seq dataset (re. 40) was reanalyzed for genes covered by the targeted gene set and expressed in ≥100 cells (indicated at the top; see Table S1 for details). Quantification was performed by counting the number of expressed genes in individual cells (binary expression).

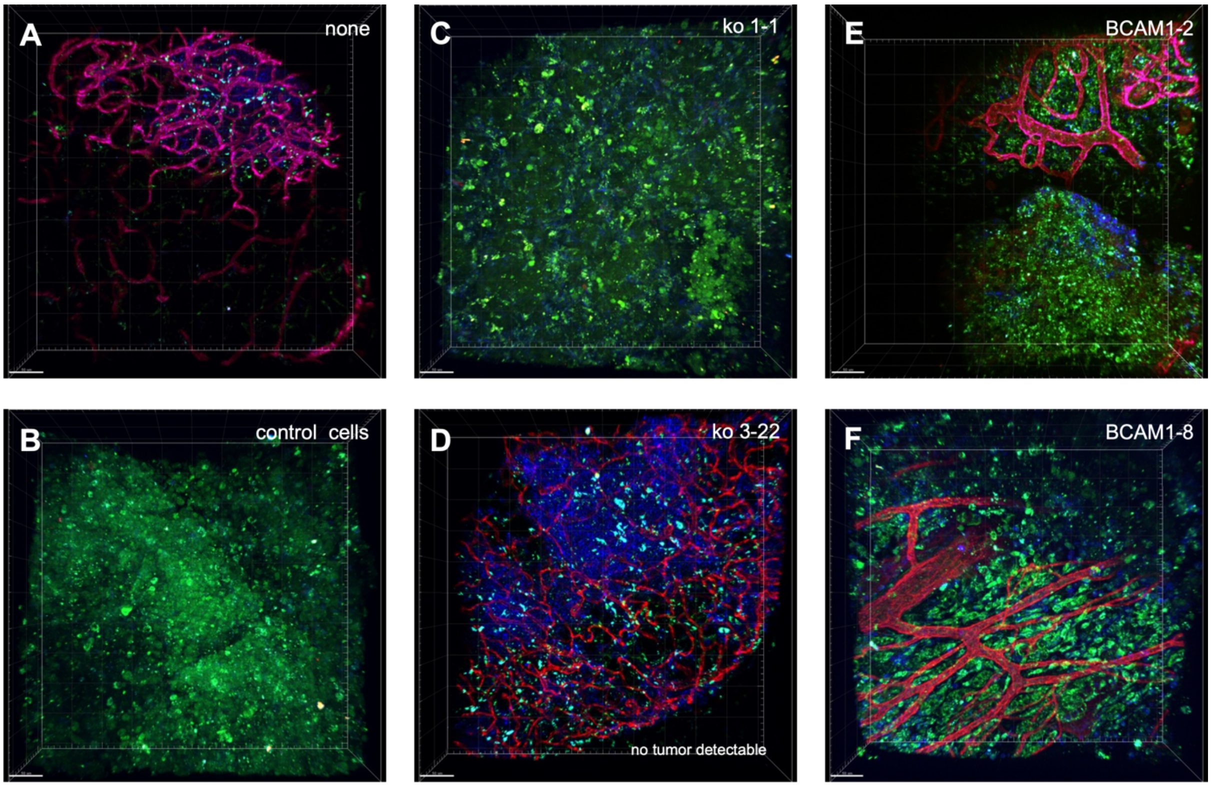

Figure S12. BCAM expression promotes the growth of blood vessels in a mouse model of peritoneal OC dissemination (case 2). The experiment was performed as in Fig. 8 (case 1). Whole-mounts of the resected omentum were stained for immune cells (CD45; blue) and blood vessels (CD31; red) and observed by multiphoton microscopy. **(A)** Representative image of the omentum from non-injected mice. **(B-F)** Omenta from mice injected i.p. with CellTracker-Green-labeled spheroids derived from the indicated cells and resected 28 days post inoculation: OVCAR-8 control cells (panel B), two different clones of OVCAR-cells with disrupted BCAM alleles (BCAM-KO-1-1, BCAM-KO-3-22; panels C and D), and or two different clones of BCAM-overexpressing OVCAR8 cells (BCAM1-2, BCAM1-8; panels E and F). Scale bar: 50 µm.

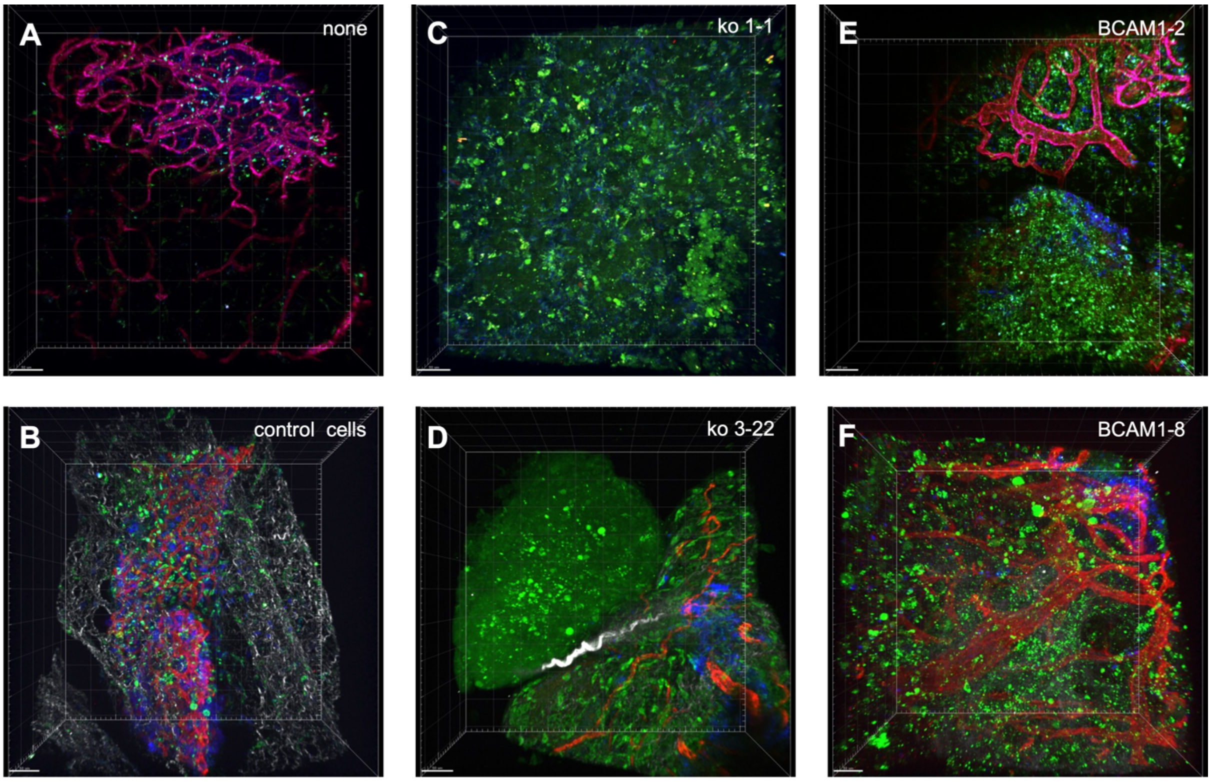

Figure S13. BCAM expression promotes the growth of blood vessels in a mouse model of peritoneal OC dissemination (case 3). See Fig. S11 for details. Scale bar: 50 µm.

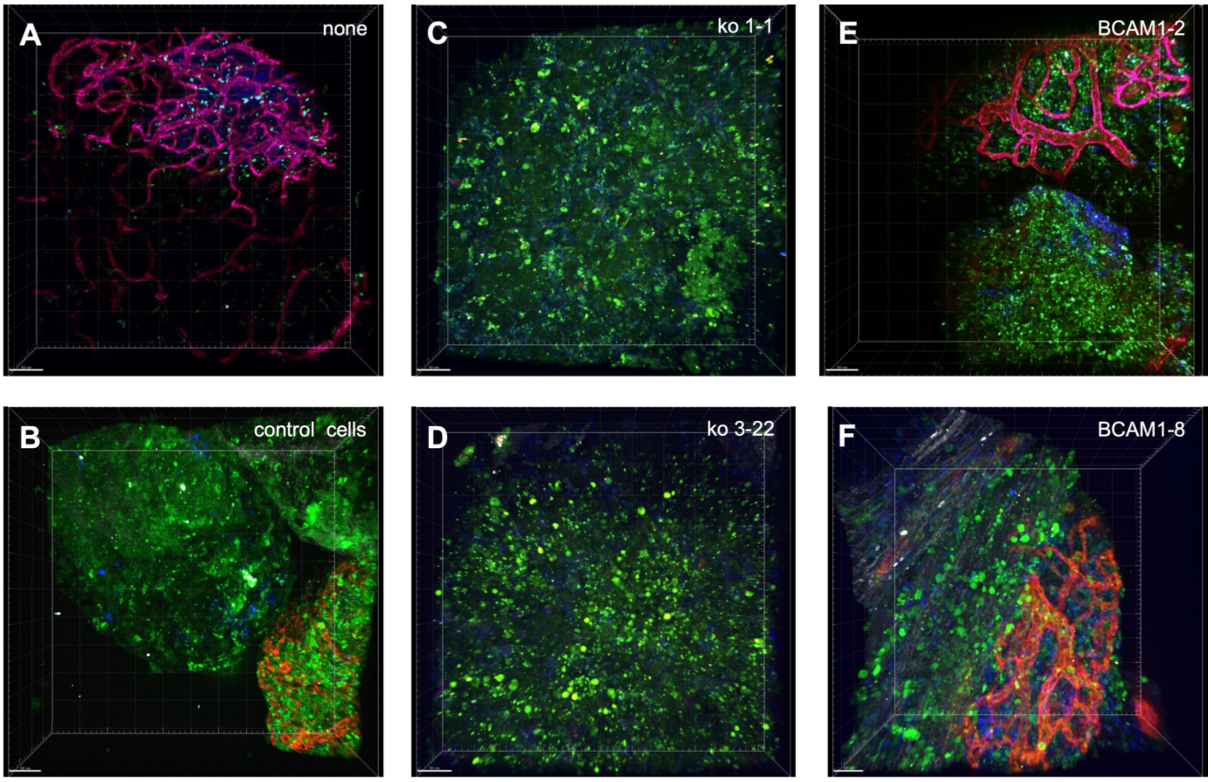

Figure S14. BCAM expression promotes the growth of blood vessels in a mouse model of peritoneal OC dissemination (case 4). See Fig. S11 for details. Scale bar: 50 µm.

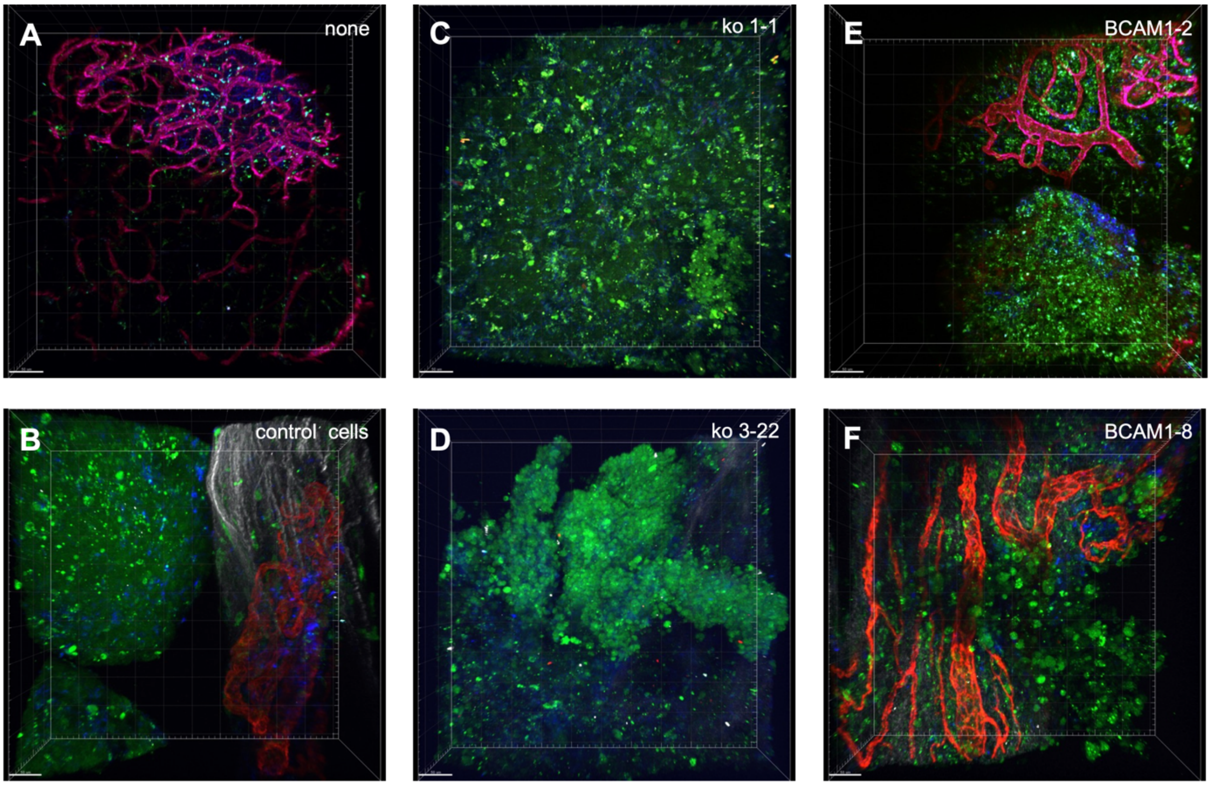

Figure S15. BCAM expression promotes the growth of blood vessels in a mouse model of peritoneal OC dissemination (case 4). See Fig. S11 for details. Scale bar: 50 µm.
